# Supplementary material for: Single cell profiling of circulating autoreactive CD4 T cells from patients with autoimmune liver diseases suggests tissue imprinting
Source: Nat Commun. 2025 Jan 29;16:1161. doi: 10.1038/s41467-025-56363-2 (PMC11779892; doi:10.1038/s41467-025-56363-2)
Supplement: Supplementary file 1 — Supplementary Information [file 41467_2025_56363_MOESM1_ESM.docx]

**Single cell profiling of circulating autoreactive CD4 T cells from patients with autoimmune liver diseases suggests tissue imprinting**

First authors : Anaïs Cardon, Thomas Guinebretière

Corresponding authors: Pierre Milpied, Amédée Renand

**List of the supplementary information**

**Supplementary figures:**

**Supplementary figure 1:** Detail of the flow cytometry gating strategy and data from three distinct patients used in the figure 1.

**Supplementary figure 2:** Characterization of the cluster 7 of PDCE2-specific CD4 T cells.

**Supplementary figure 3:** Longitudinal analysis of Sepsecs-specific clonotypes.

**Supplementary figure 4:** Literature-based-gene set score analysis of cells from clusters identified in the figure 2F.

**Supplementary figure 5:** Spatial multi-phenotyping analysis on a liver biopsy from one AIH patient.

**Supplementary figure 6:** Spatial lineage assignment strategy and proximity analysis.

**Supplementary figure 7:** Sepsecs-clonotypes are enriched in the liver of an SLA^+^ patient.

**Supplementary figure 8:** Sorting strategy of blood PD-1^+^ or PD-1^-^ CD4 T cells.

**Supplementary figure 9:** Shared TCRβ sequences between Blood PD-1^+^ or PD-1^-^ CD4 T cells and the liver biopsies from four distinct patients.

**Supplementary figure 10:** Gene signature of PD-1^+^CXCR5^-^ memory CD4 T cells clusters.

**Supplementary figure 11:** Unsupervised characterization of PD-1^+^CXCR5^-^ memory CD4 T cell subsets.

**Supplementary figure 12:** Literature-based-gene set score analysis of cells from clusters identified in the figure 3.

**Supplementary figure 13:** Sorting strategy of blood Tetramer^+^ CD4 T cells.

**Supplementary figure 14:** Literature-based-gene set score analysis of Tetramer positive or negative cells identified in the figure 4.

**Supplementary figure 15:** Unsupervised flow cytometry analysis.

**Supplementary figure 16:** Supervised identification of the cluster 15 (PD-1^+^CXCR5^-^TIGIT^+^HLA-DR^+^).

**Supplementary figure 17:** Intracellular characteristics of PD-1^+^CXCR5^-^TIGIT^+^ memory CD4 T cells.

**Supplementary figure 18:** Sorting strategy of blood memory CD4 T cell subsets, presented in the Figure 5.

**Supplementary Figure 19:** Tamoxifen treatment in non-TCR-transgenic mouse model**.**

**Supplementary Figure 20:** Flow cytometry gating strategy for analysis of HA tetramer-specific CD4 and CD8 T cells in mice spleen and liver.

**Supplementary Figure 21:** Analysis of IFNγ-secreting HA-specific CD4 and CD8 T cells after immune checkpoint blockade.

**Supplementary tables:**

**Supplementary Table 1**. Clinical and biological characteristics of patients with AIH and NASH expressed as mean [95% confidence interval] in the figure 5.

**Supplementary Table 2.** Antibody listing.

**Supplementary Table 3:** Oligonucleotides listing.

**Supplementary figures:**

**
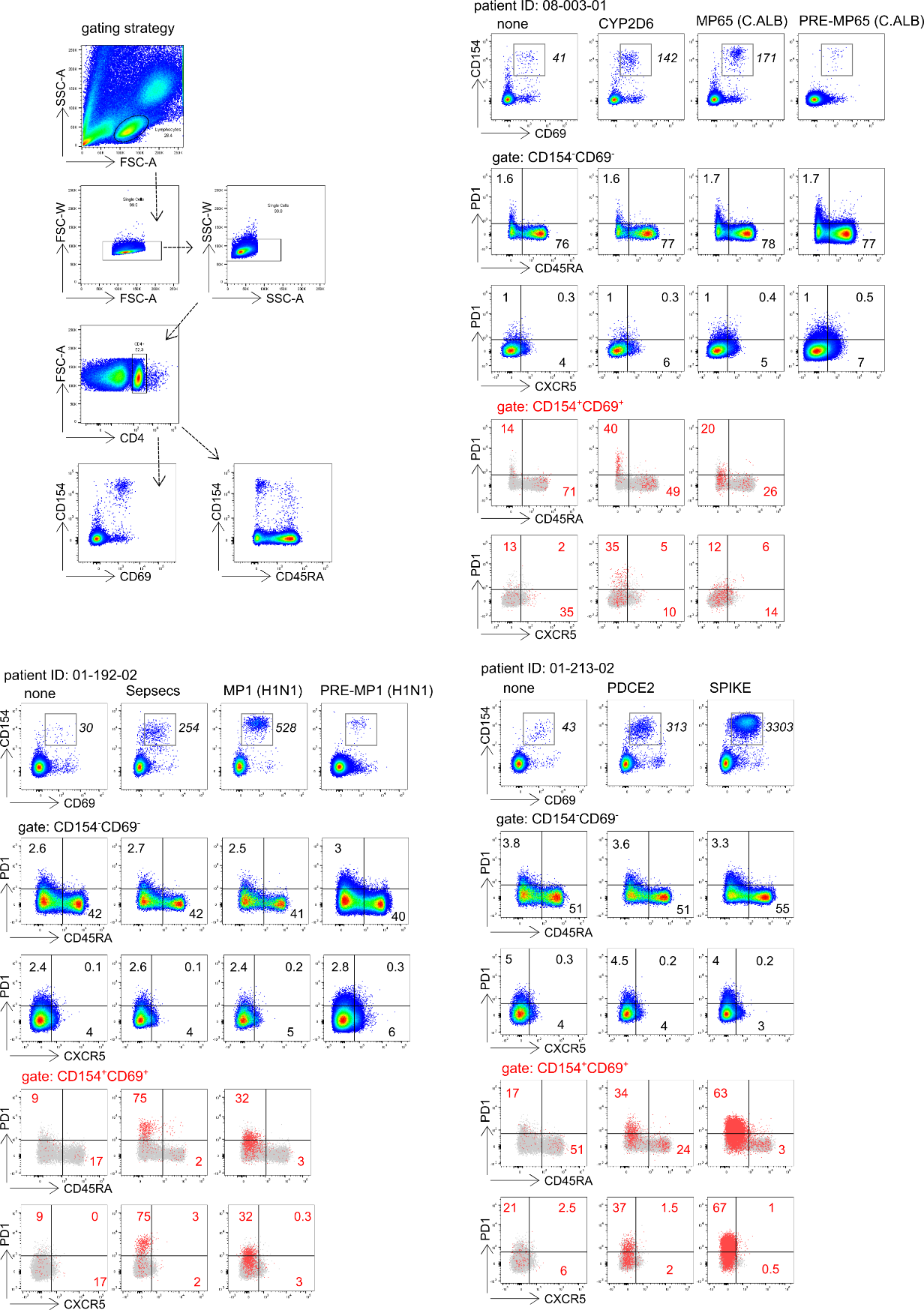
**

**Supplementary figure 1: Detail of the flow cytometry gating strategy and data from three distinct patients used in the figure 1.** Number in italic represent the number of CD69^+^CD154^+^ cells per million total CD4. Number in each quadrants represent percentages.


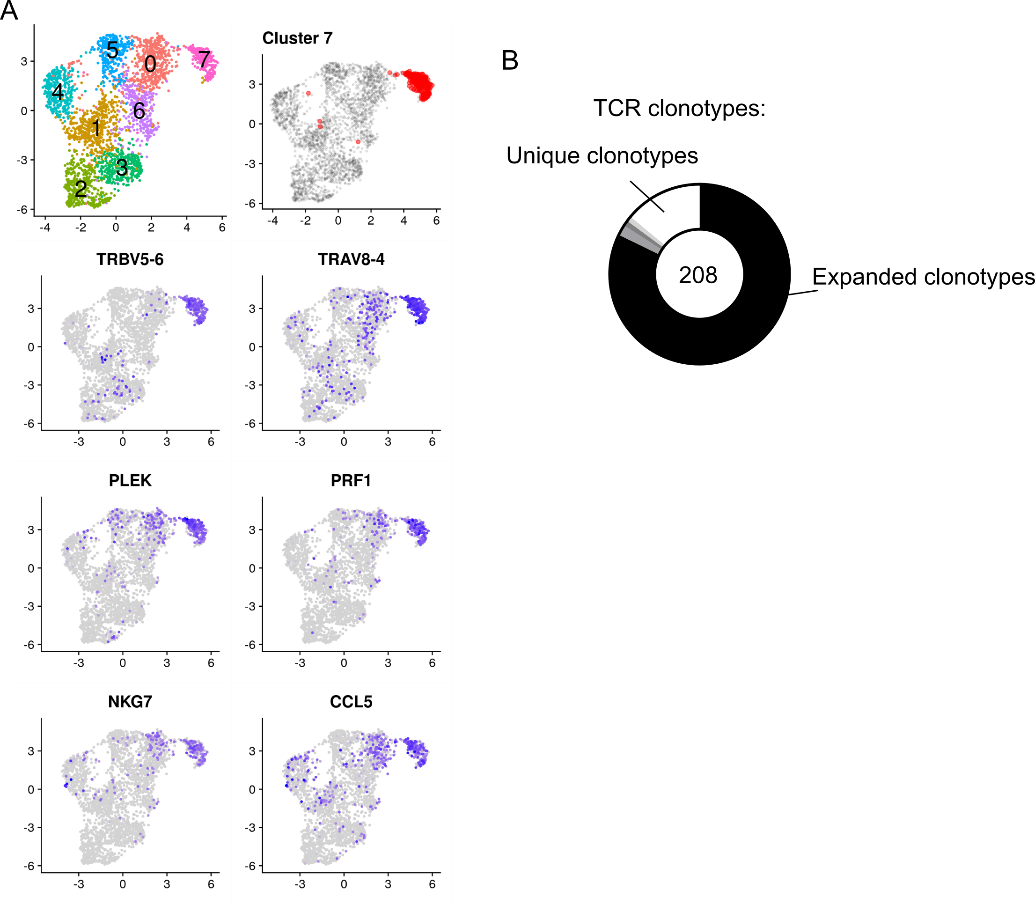


**Supplementary figure 2: Characterization of the cluster 7 of PDCE2-specific CD4 T cells.** (A) UMAP representation of selected gene markers. (B) TCRαβ clonal diversity of antigen-specific single T cells for cluster 7 cells. Numbers indicate the number of single cells analyzed with a TCRαβ sequence. Black and grey sectors indicate the proportion of TCRαβ clones (clonotype common to ≥ 2 cells) within single-cells analyzed; white sector: unique clonotypes.


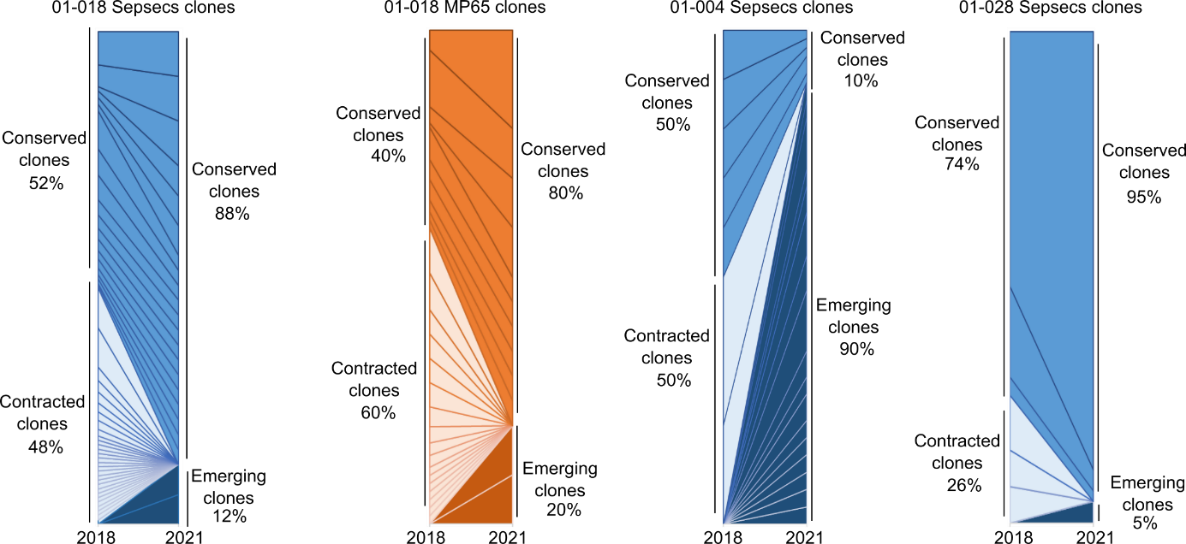


**Supplementary figure 3: Longitudinal analysis of Sepsecs-specific clonotypes.** Representation of the proportion of Sepsecs- and MP65-clonotypes between 2018 and 2021 in the blood of three patients.


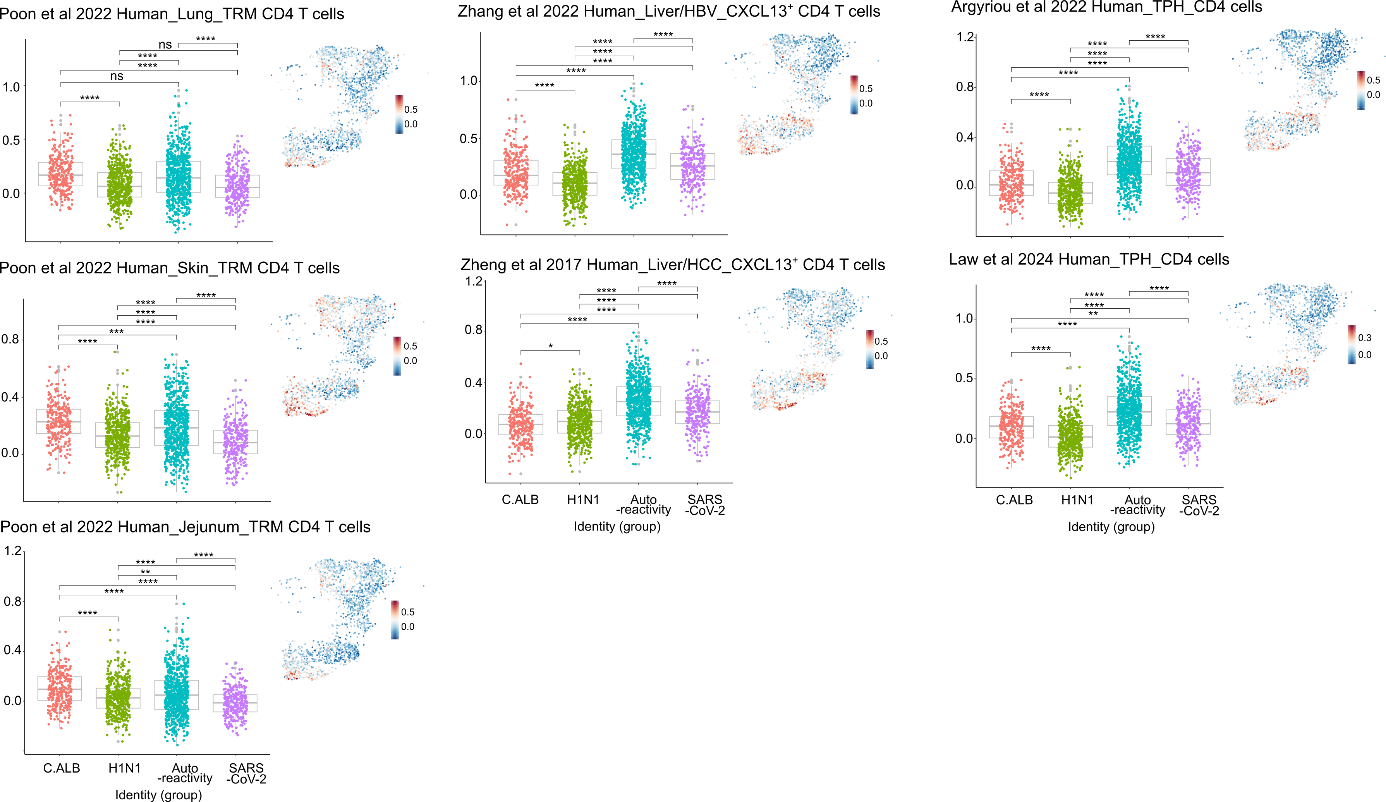


**Supplementary figure 4: Literature-based-gene set score analysis of cells from clusters identified in the figure 2F.** Two-sided, pairwise comparison with Benjamini-Hochberg corrected Wilcoxon test. *: p<0.05; **: p<0.01; ***: p<0.001; ****: p<0.0001. ns: non-significant. P values are listed in the supplementary Data 5. Data are presented as mean values ± SD. Source data are provided as a Source Data file.

**
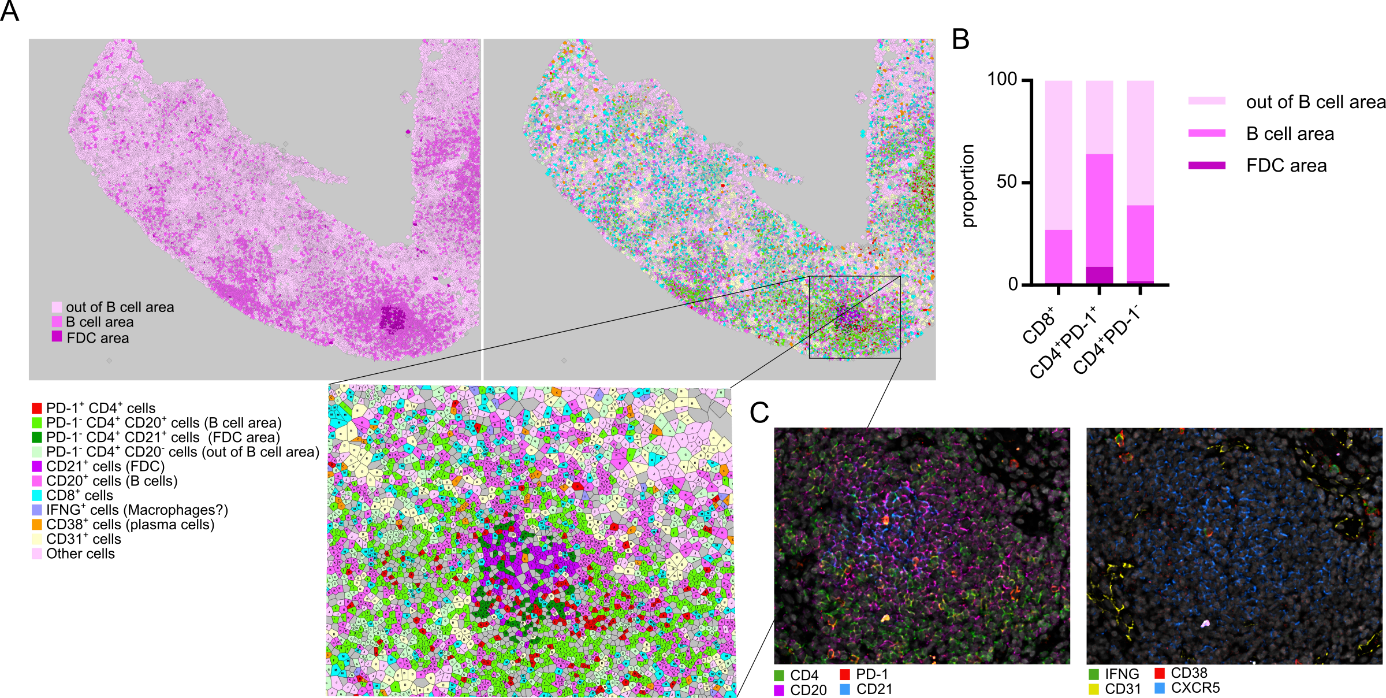
**

**Supplementary figure 5: Spatial multi-phenotyping analysis on a liver biopsy from one AIH patient.** (A) Voronoi representation of cellular subsets in an AIH liver biopsy after cell segmentation. FDC area is defined by expression of CD21; B cell area is defined by CD20 expression; and out of B cell area is defined by the absence of CD20 expression. The lower panel shows a zoom on the selection indicated in the upper panel. (B) Representation of the repartition of CD8^+^, CD4^+^ PD-1^+^ and CD4^+^ PD-1^-^ cells in FDC, B cell and out of B cell area defined in (A). (C) CD4, CD20, PD-1, CD21, IFNγ, CD38, CD31 and CXCR5 marker staining within segmentation.


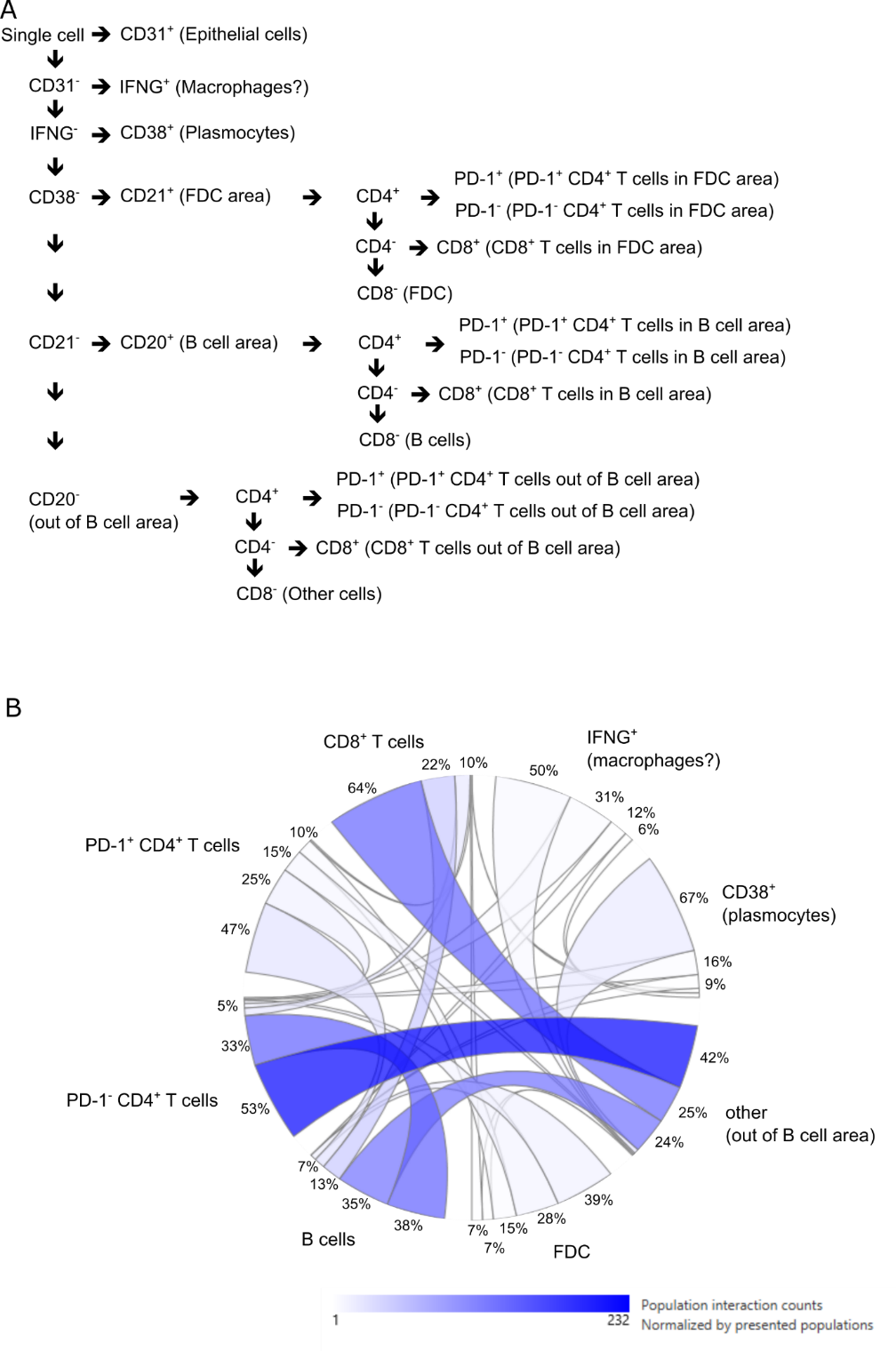


**Supplementary figure 6: Spatial lineage assignment strategy and proximity analysis.** (A) Schematic lineage assignment strategy. (B) Proximity analysis between major cellular subsets identified. Analysis performs with the Multiple Analysis Viewer (MAV) software.


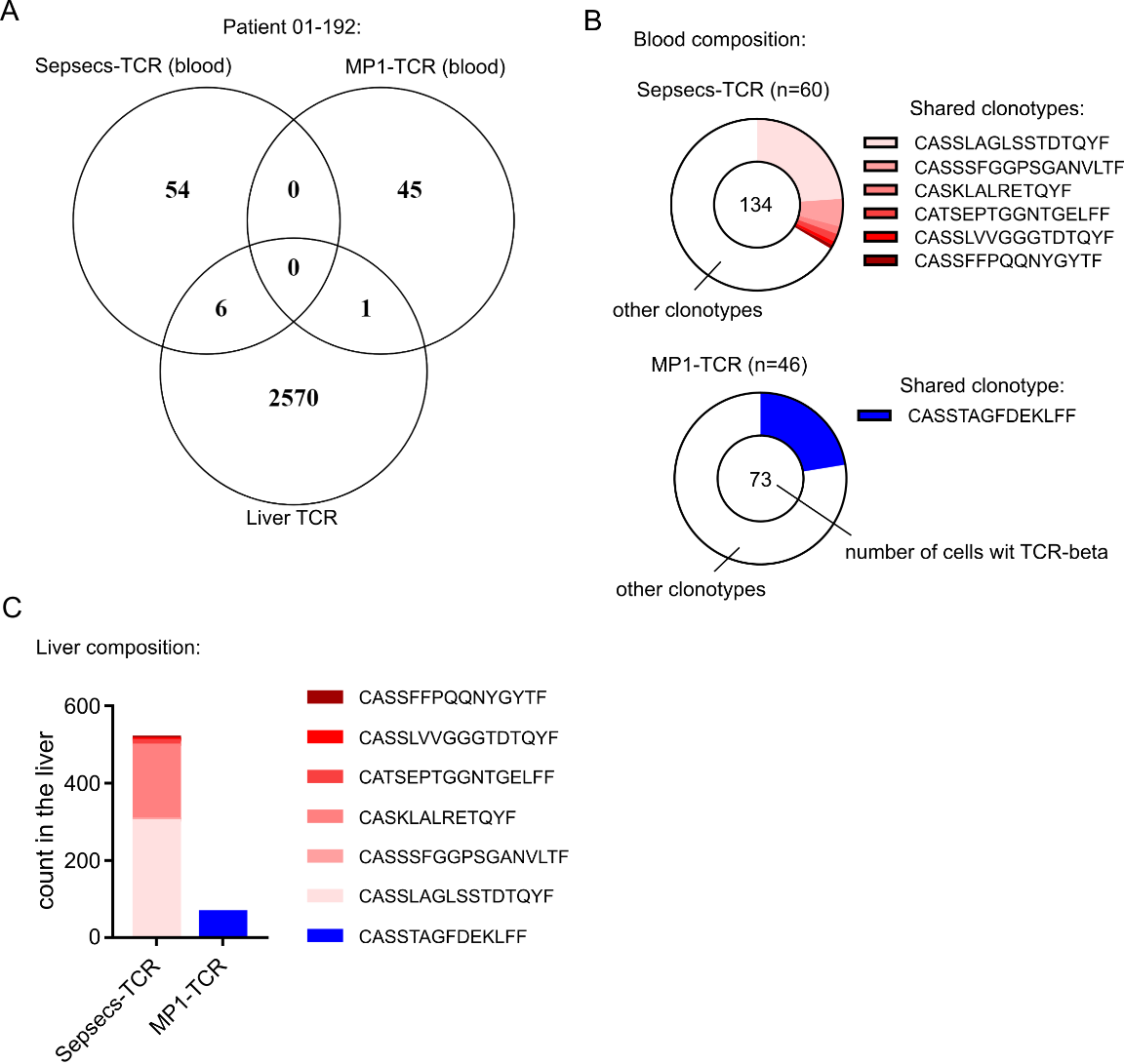


**Supplementary figure 7: Sepsecs-clonotypes are enriched in the liver of an SLA^+^ patient.** (A) Shared TCR between Sepsecs-specific CD4 T cells, MP1-specific CD4 T cells and the liver biopsy. (B) Proportion of shared TCR with the liver (colored sectors) within TCRαβ clonal diversity of Sepsecs- or MP1-specific CD4 T cells. (C) Liver frequency (count) of shared-TCRβ sequences.


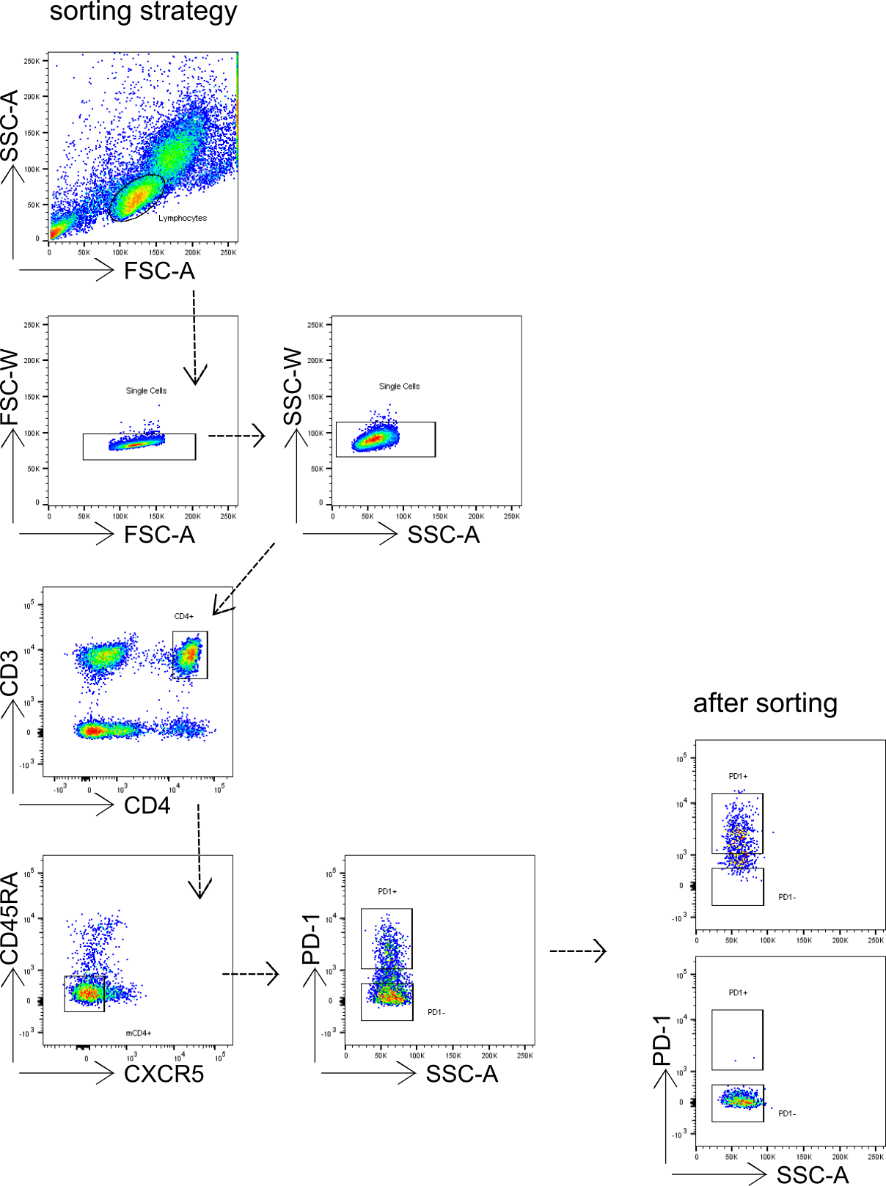


**Supplementary figure 8: Sorting strategy of blood PD-1^+^ or PD-1^-^ CD4 T cells.** T cell sorting from frozen PBMCs from AIH patients, using a panel of 5 colored antibodies (CD3, CD4, CD45RA, CXCR5 and PD-1) and BD FACSAriaII sorter.


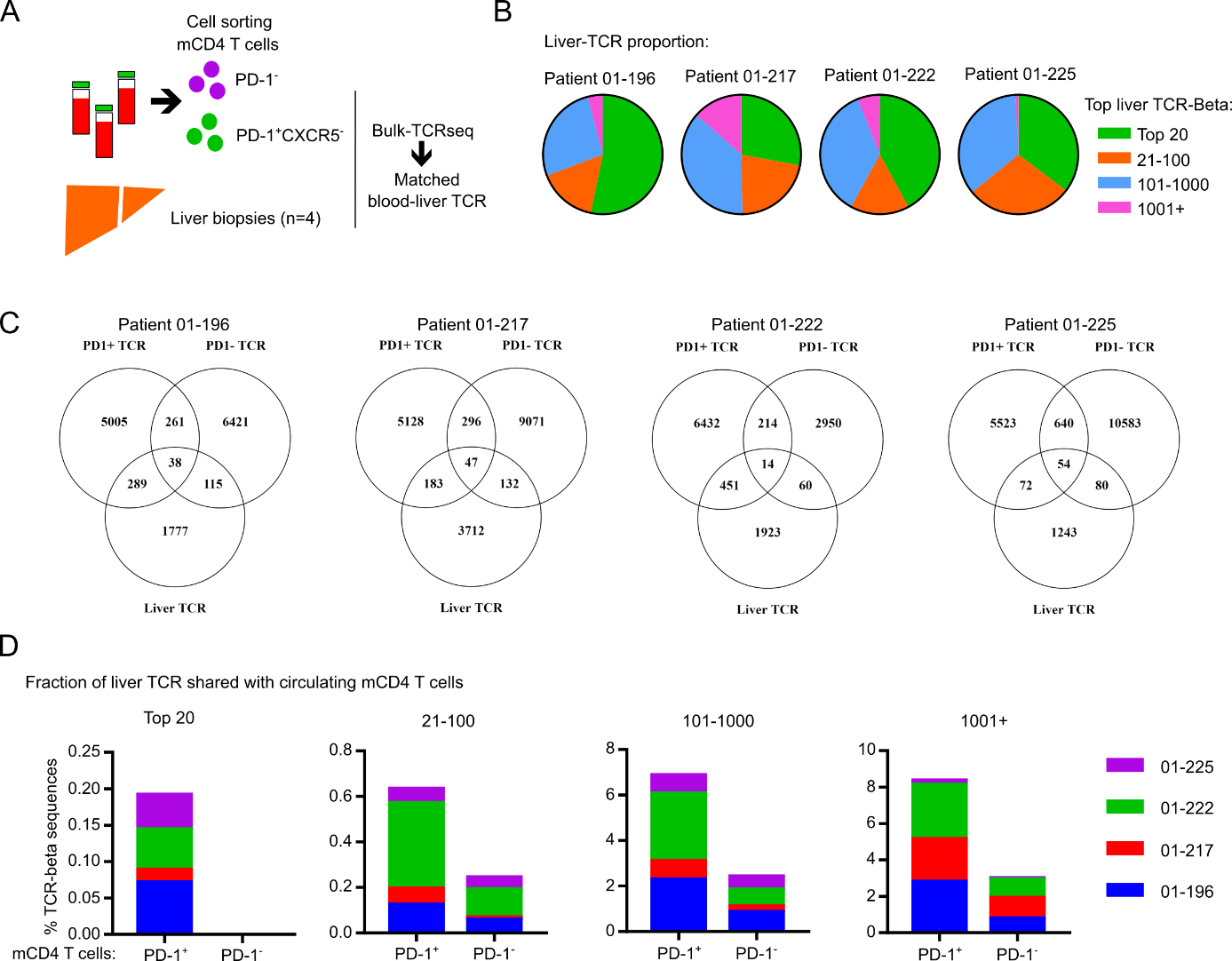


**Supplementary figure 9: Shared TCRβ sequences between Blood PD-1^+^ or PD-1^-^ CD4 T cells and the liver biopsies from four distinct patients**. (A) Experimental design for bulk TCRβ sequencing. (B) Pie chart showing the proportion of the TCRβ repertoire occupied by the top 20, 100, 1000 or 1001+ clones. (C) Shared TCRβ sequences between Blood PD-1^+^ or PD-1^-^ mCD4 T cells and the liver biopsies from the four distinct patients. (D) Analysis of the percentage of TCRβ clonotypes shared between the top 20, 100, 1000 or 1001+ liver clones and the circulating PD-1^+^CXCR5^-^ (PD-1^+^) or PD-1^-^ mCD4 T cells (PD-1^-^) from four distinct AILD patients. Source data are provided as a Source Data file.


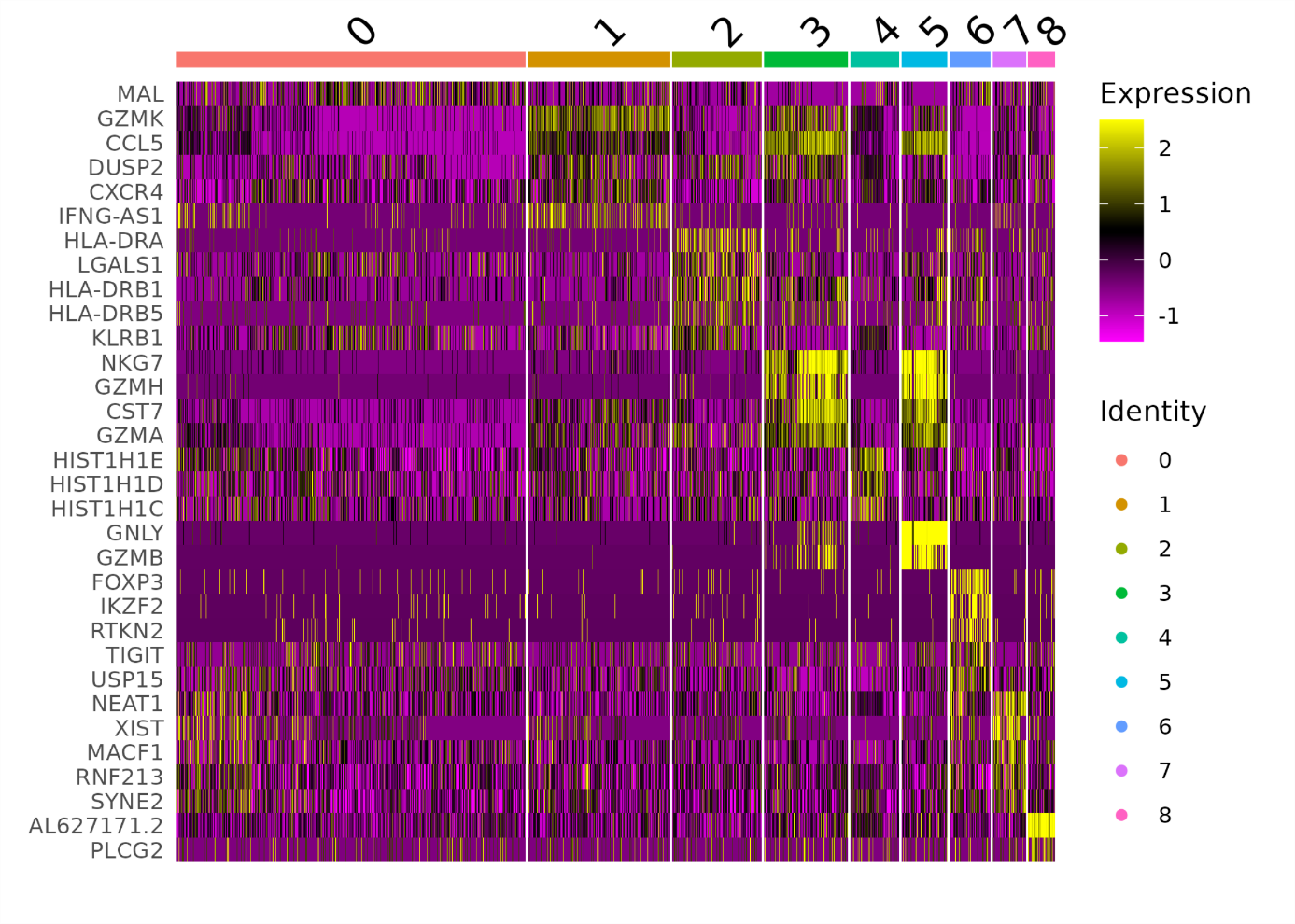


**Supplementary figure 10: Gene signature of PD-1^+^CXCR5^-^ memory CD4 T cells clusters.** Single cell gene expression heatmap for top max 5 marker genes of PD-1^+^CXCR5^-^ memory CD4 T cells clusters.


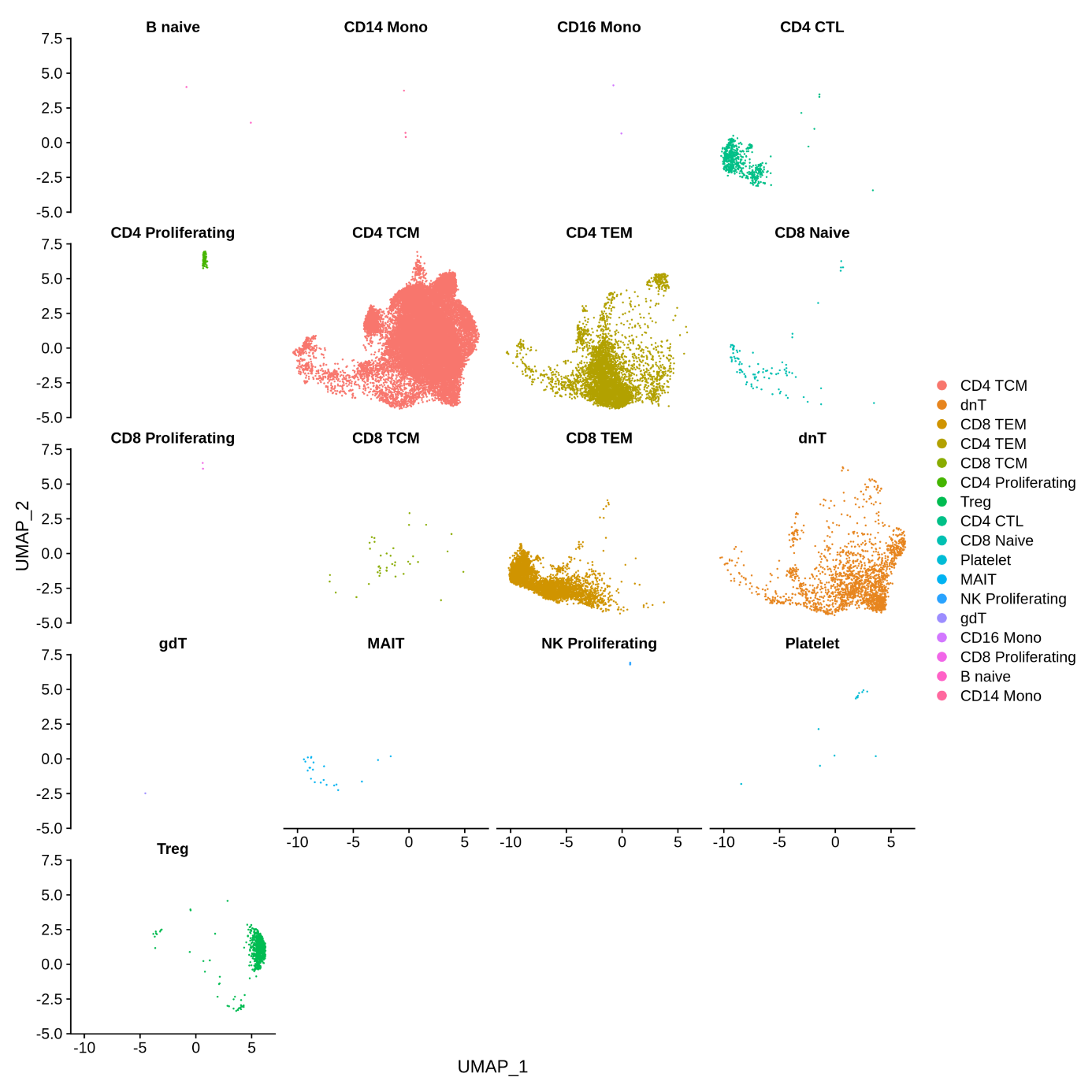


**Supplementary figure 11: Unsupervised characterization of PD-1^+^CXCR5^-^ memory CD4 T cell subsets.** Unsupervised azimuth annotation of scRNA-seq data from PD-1^+^CXCR5^-^ memory CD4 T cell subsets.

**
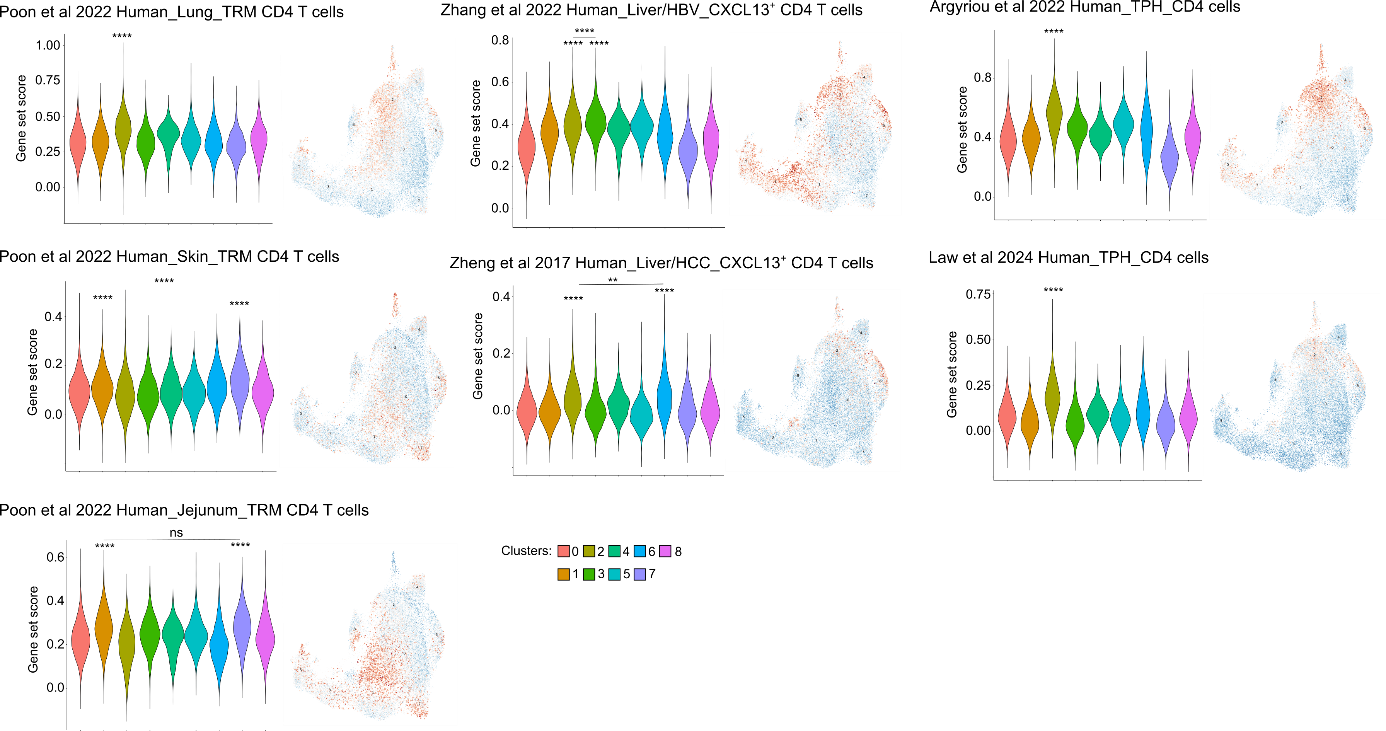
**

**Supplementary figure 12: Literature-based-gene set score analysis of cells from clusters identified in the figure 3.** Two sided, pairwise comparison with Kruskal Wallis test followed by a paired Wilcoxon rank test. **: p<0.01; ****: p<0.0001. ns: non-significant. All p values are listed in the supplementary Data 9. Source data are provided as a Source Data file.

**
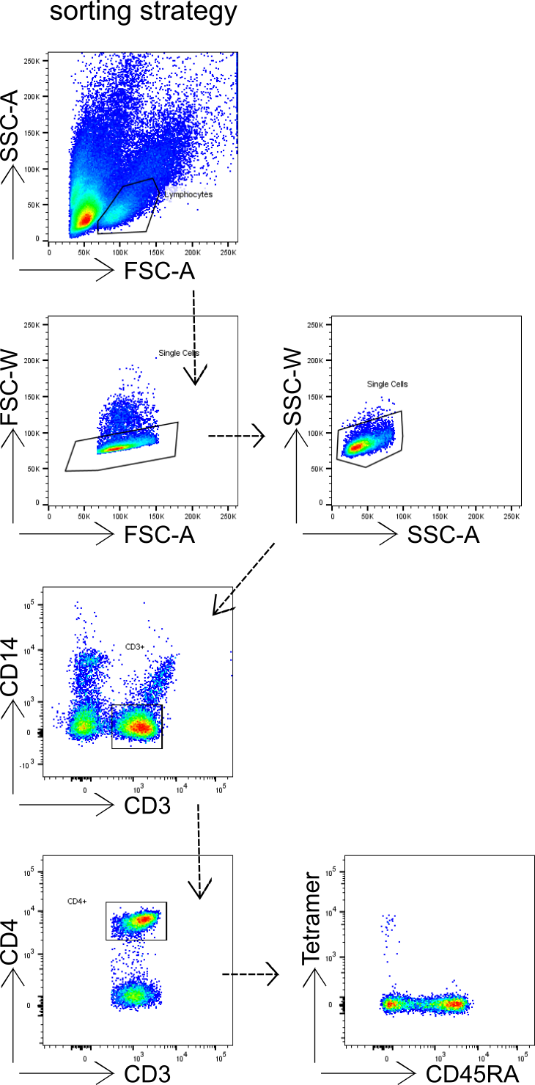
**

**Supplementary figure 13: Sorting strategy of blood Tetramer^+^ CD4 T cells.** T cell sorting from frozen PBMCs from AIH patients, using a panel of 6 colored antibodies (CD14, CD3, CD4, CD45RA, PD-1 and Tetramer) and BD FACSAriaII sorter.

**
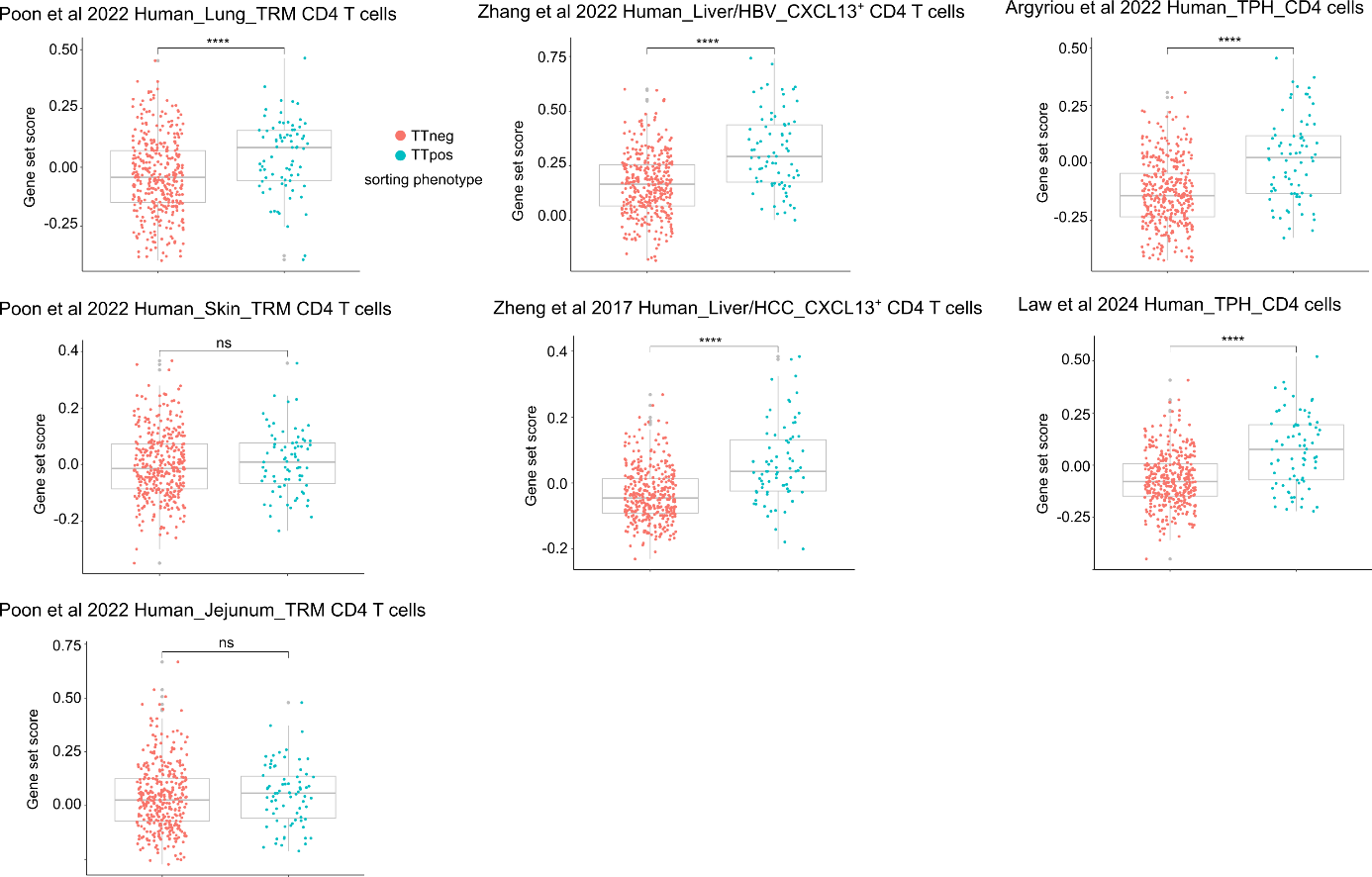
**

**Supplementary figure 14: Literature-based-gene set score analysis of Tetramer positive or negative cells identified in the figure 4.** Two sided, unpaired Mann-Whitney test. Data are presented as mean values ± SD. Source data are provided as a Source Data file. Poon_et_al_2022_Human_Lung, p=0.000021. Poon_et_al_2022_Human_Skin, p=0.41. Poon_et_al_2022_Human_jejunum, p=0.24. Zhang_2022_Human_Liver_HBV, p=9.2x10^-11^. Zheng_2017_Human_HCC, p=1.1x10^-11^. Argyriou_2022_Human_TPH, p=1.4x10^-10^. Law_et_al_2024_Human_ TPH, p=5.1x10^-10^.


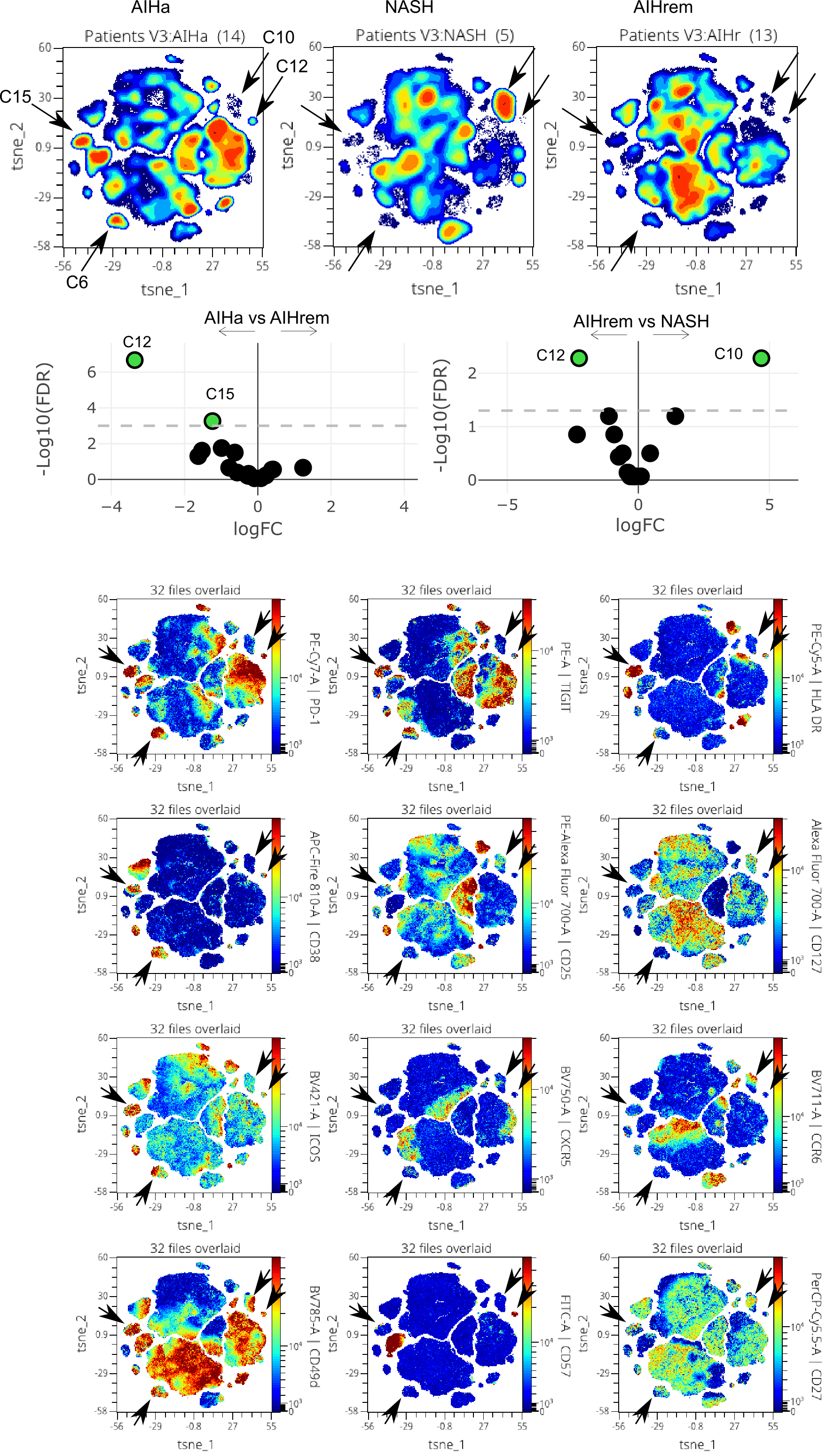


**Supplementary figure 15: Unsupervised flow cytometry analysis**. Detailed unsupervised flow cytometry analysis of active AIH (AIHa), NASH and AIH in remission (AIHrem) patients and expression of indicated markers on the opt-SNE representation of blood memory CD4 T cell subsets.


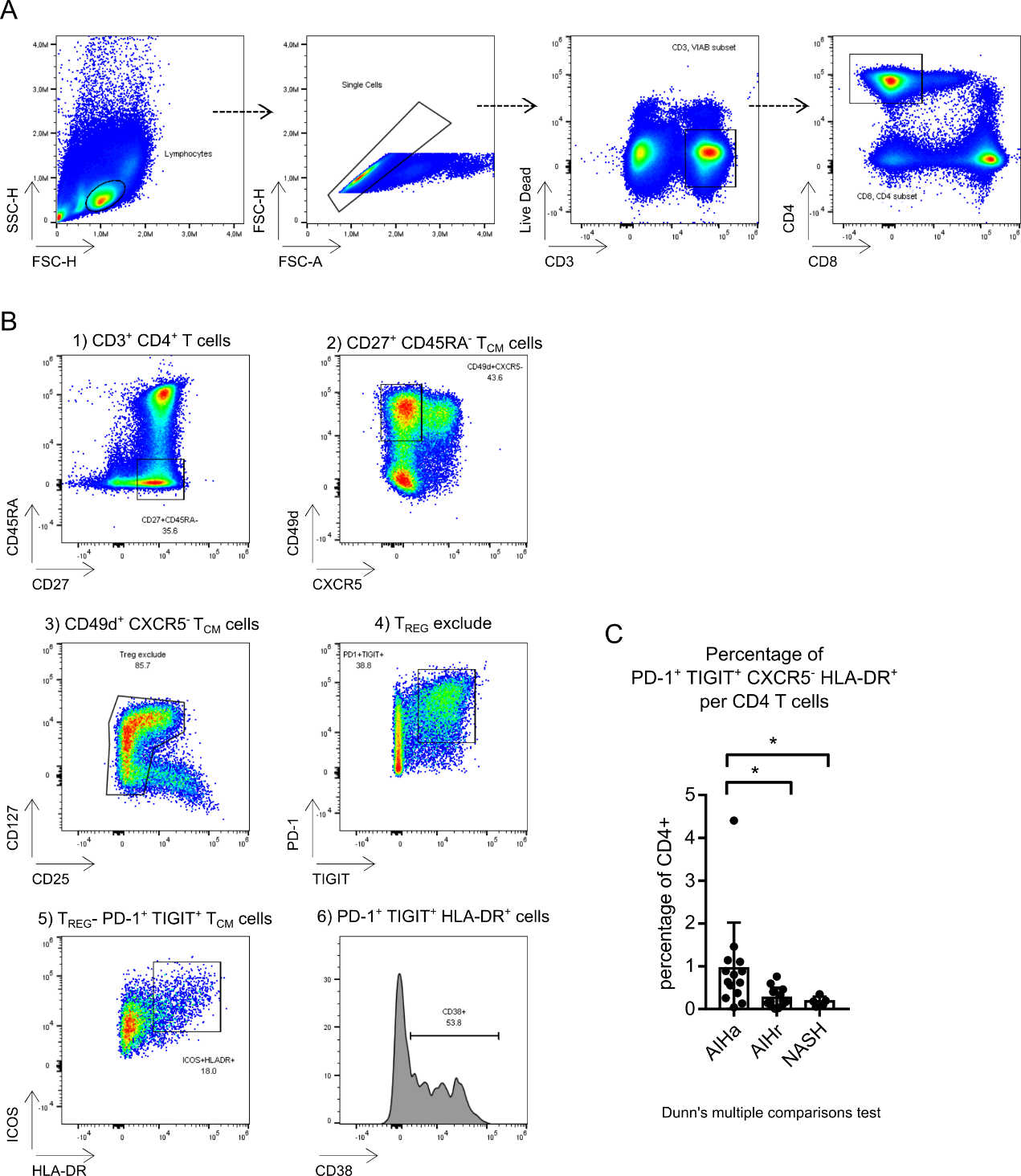


**Supplementary figure 16: Supervised identification of the cluster 15 (PD-1^+^CXCR5^-^TIGIT^+^HLA-DR^+^).** (A) Gating strategy to isolate CD3^+^CD4^+^ T cells. (B) Dot plot representation of the gating strategy. (C) Frequency of PD-1^+^ CXCR5^-^ TIGIT^+^ HLA-DR^+^ CD4 T cells per total CD4^+^ T cells in the blood of 5 NASH, 14 active AIH (AIHa), and 13 AIH patients in remission (AIHr) under treatment (<2 years, n=4; >2years, n=12). AIHa vs AIHr: p=0.0154; AIHa vs NASH: p=0.0399; AIHr vs NASH: p>0.9999. Frozen PBMCs were analyzed using a panel of 21 colored antibodies (CXCR4, ICOS, CD200, CXCR3, CCR6, CXCR5, CD49d, CD57, TIGIT, HLA-DR, CD25, CD27, CD127, CD38, CD45RA, CD16, CD56, CD8, CD3, CD161, PD-1 and CD4) and Cytek spectral flow cytometer Aurora. Data are presented as mean values ± SD. Two-sided, one way ANOVA, Kruskal-Wallis test and Dunn's multiple comparisons test for C. Source data are provided as a Source Data file.


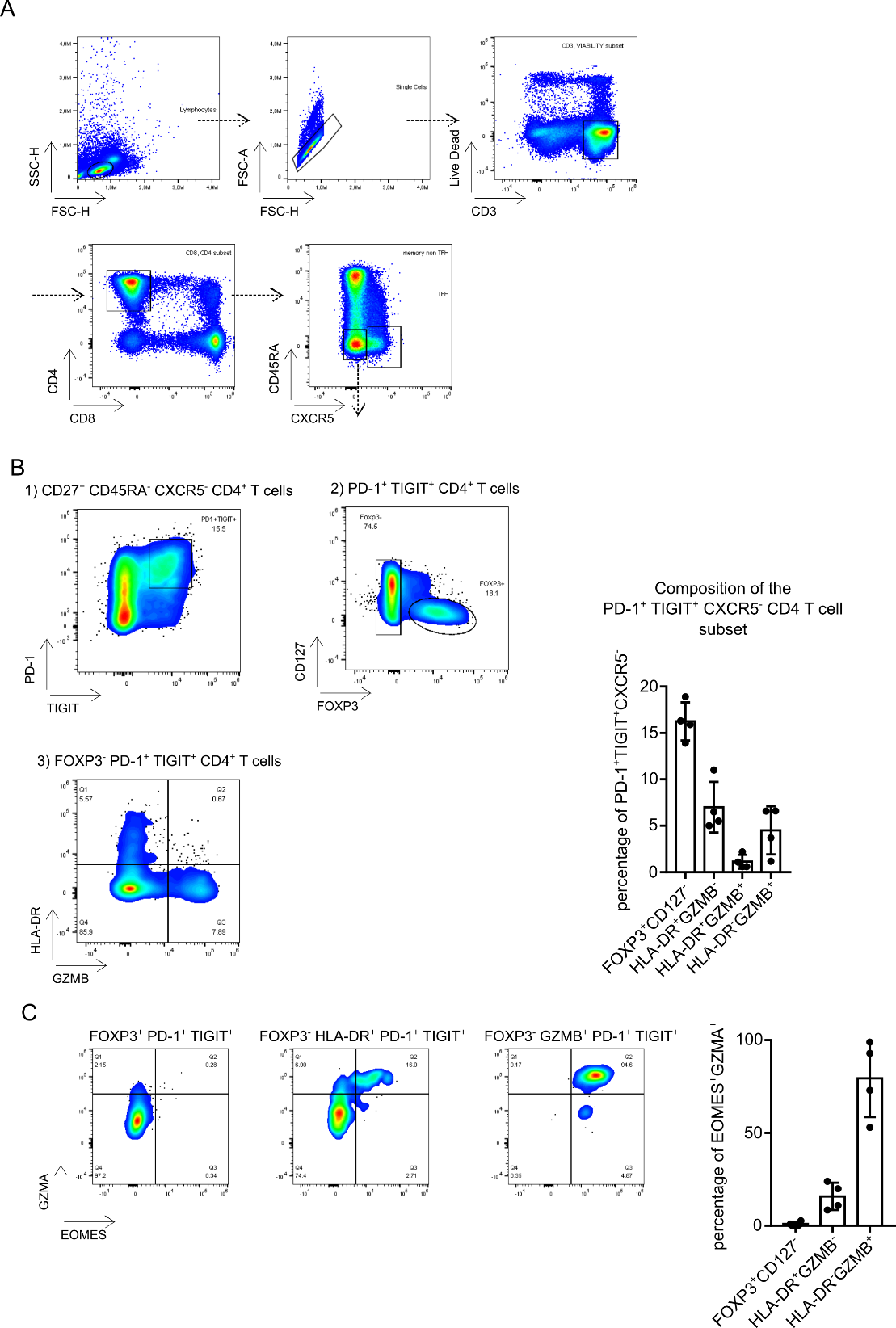


**Supplementary figure 17: Intracellular characteristics of PD-1^+^CXCR5^-^TIGIT^+^ memory CD4 T cells.** (A) Gating strategy. (B) Foxp3, Granzyme B (GZMB) and HLA-DR expression within PD-1^+^CXCR5^-^TIGIT^+^ memory CD4 T cells. (B) Eomes and GZMA expression by PD-1^+^CXCR5^-^TIGIT^+^FOXP3^+^ (FOXP3^+^CD127^-^); PD-1^+^CXCR5^-^TIGIT^+^FOXP3^-^HLA-DR^+^ (HLA-DR^+^GZMB^-^) and PD-1^+^CXCR5^-^TIGIT^+^GZMB^+^ memory CD4 T cells (HLA-DR^-^GZMB^+^). Frozen PBMCs were analyzed using a panel of 18 colored antibodies (TIGIT, HLA-DR, CD127, CD38, CD45RA, CD16, CD56, CD8, CD3, CD161, PD-1, CD4, CD27, Granzyme B, CD28, CD39, EOMES, FOXP3) and Cytek spectral flow cytometer Aurora. Data are presented as mean values ± SD. Source data are provided as a Source Data file.


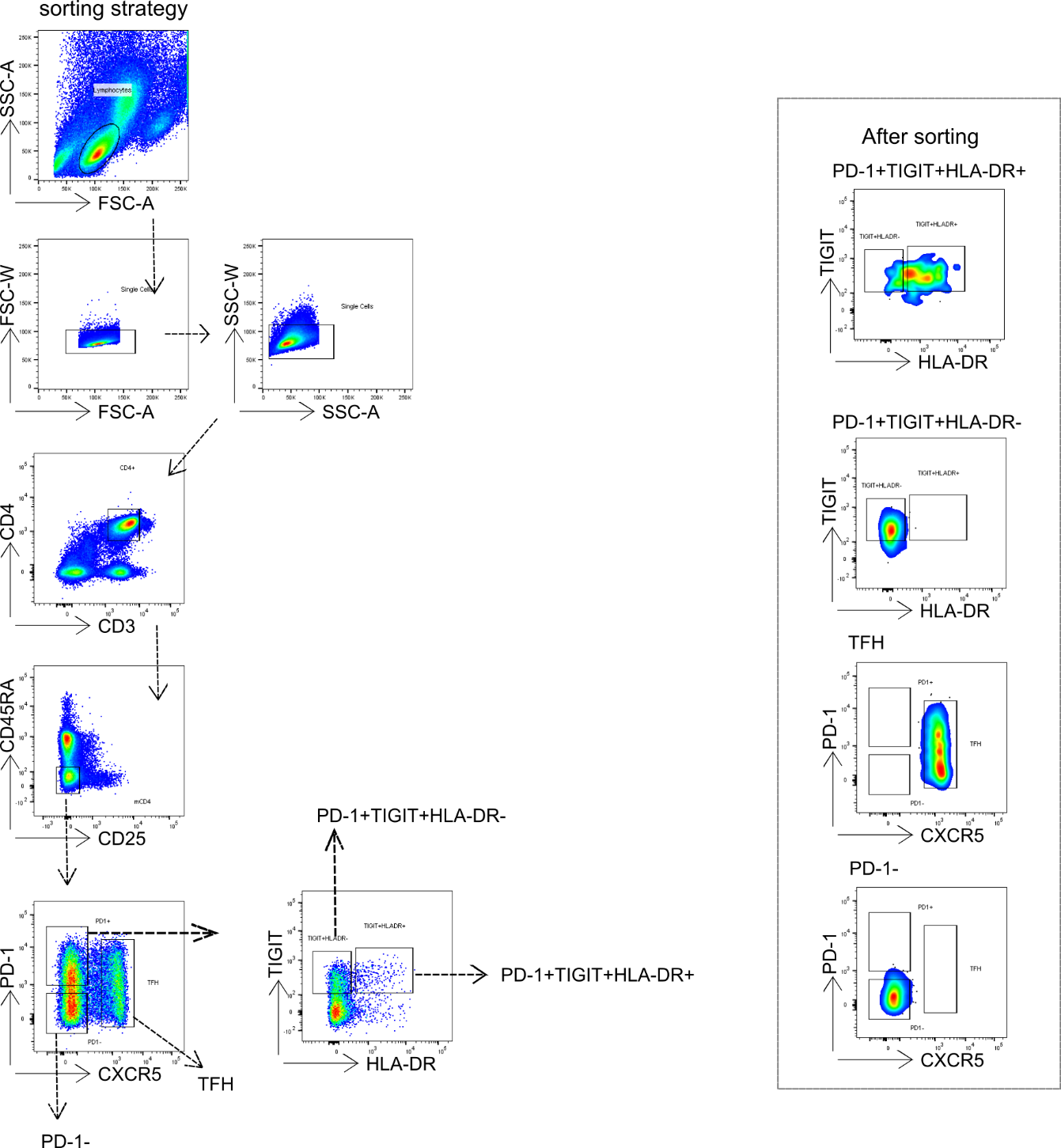


**Supplementary figure 18: Sorting strategy of blood memory CD4 T cell subsets, presented in the Figure 5.** T cell sorting from fresh PBMCs of AIH patients, using a panel of 8 colored antibodies (CD3, CD4, CD45RA, CD25, PD-1, CXCR5, TIGIT and HLA-DR) and BD FACSAriaII.


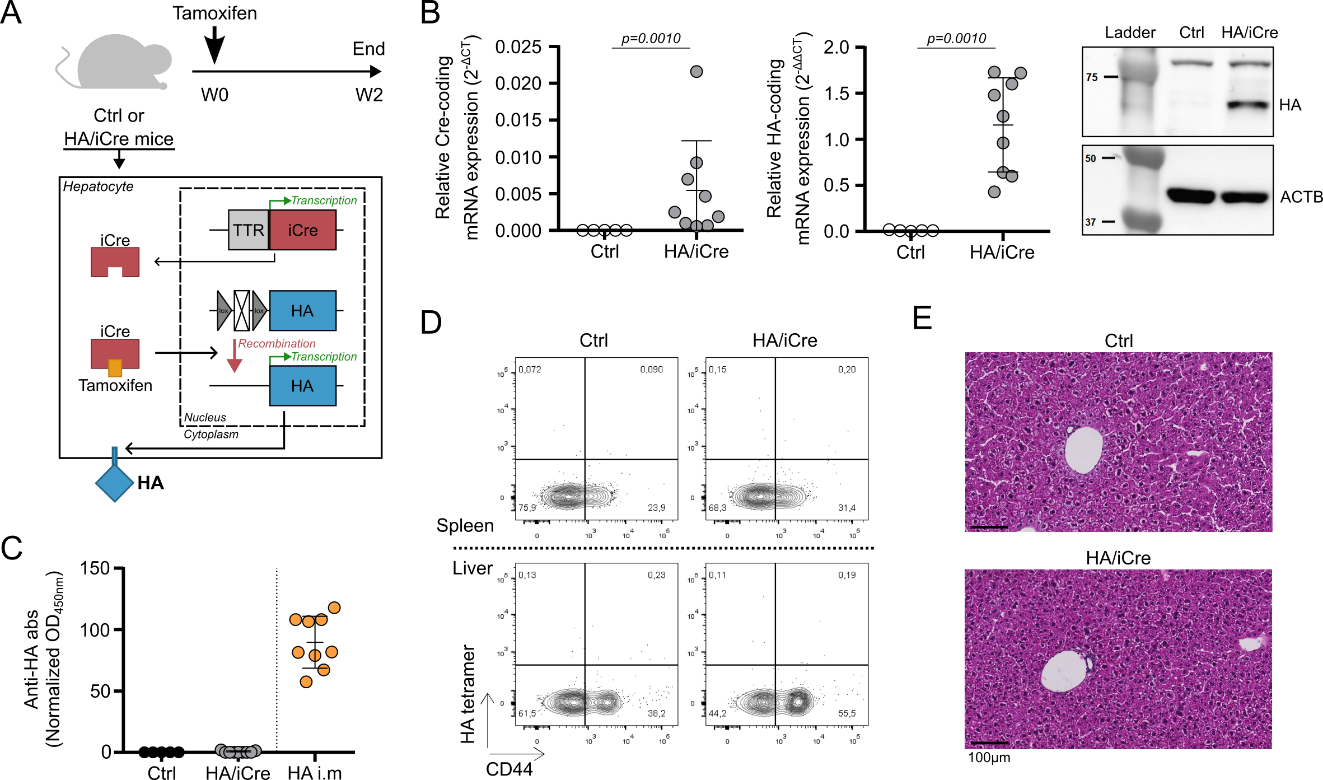


**Supplementary Figure 19: Tamoxifen treatment in non-TCR-transgenic mouse model.** (A) Experimental design and schematic illustration for tamoxifen treatment in Rosa26 HA floxed mice (Ctrl, n=5) and Rosa26 HA floxed TTR-inducible Cre mice (HA/iCre, n=9). (B) Relative Cre mRNA expression (left) and relative HA mRNA expression (middle) in the liver. HA protein detection from total protein extract of liver samples (right). ACTB was used as loading control. (C) Analysis of normalized anti-HA antibody rate in serum of tamoxifen-treated Ctrl and HA/iCre mice. Anti-HA antibody rate of immunized mice (HA i.m) was indicated as positive control. (D) Contour plot representation of HA tetramer staining and CD44 expression in CD4 T cells from spleen (top) and liver (bottom) of tamoxifen-treated Ctrl and HA/iCre mice. (E) Representative pictures of paraffin-embedded liver sections stained with HPS coloration. Black line is used as scale. Data are presented as mean values ± SD in graphs B and C. Two-sided Mann-Whitney test was used for B. p-values are indicated. Source data are provided as a Source Data file.


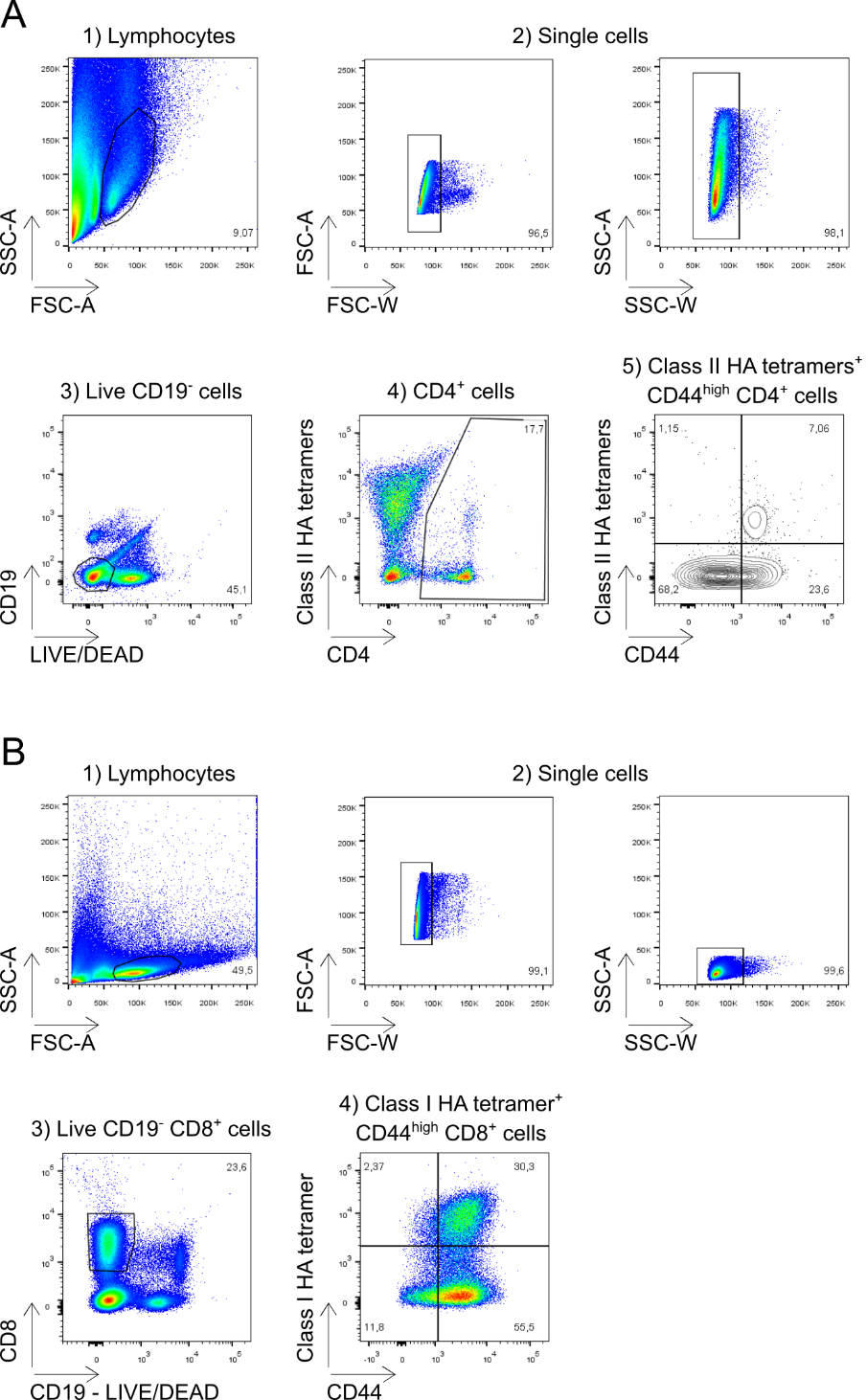


**Supplementary Figure 20: Flow cytometry gating strategy for analysis of HA tetramer-specific CD4 and CD8 T cells in mice spleen and liver.** (A) Dot plot representation of gating strategy for analysis of HA tetramer-specific CD4 T cells. (B) Dot plot representation of gating strategy for analysis of HA tetramer-specific CD8 T cells.


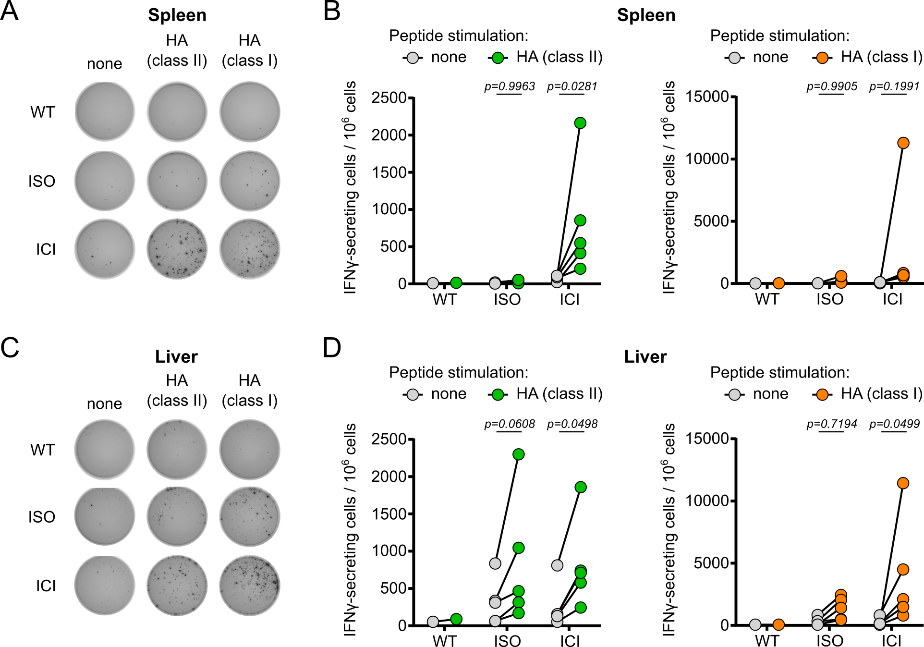


**Supplementary Figure 21: Analysis of IFNγ-secreting HA-specific CD4 and CD8 T cells after immune checkpoint blockade.** (A) Representative pictures of HA-specific spot-forming assays on splenocytes. Each black spot indicates one IFNγ-secreting cell. Splenocytes from wild-type mouse (WT, n=1), tamoxifen-treated HA immunized HA/iCre mice that received isotype control antibodies (ISO, n=5) or anti-PD-1/CTLA-4 blocking antibodies (ICI, n=5) were stimulated with CD4-stimulating HA peptides (class II) or CD8-stimulating HA peptide (class I) or non-stimulated (none). (B) Frequency of IFNγ-secreting cells per million cells after HA class II (left) and HA class I stimulation (right). (C) Representative pictures of HA-specific spot-forming assays on Liver non-parenchymal cells (NPCs). Each black spot indicates one IFNγ-secreting cells. Liver NPCs from WT, ISO and ICI mice were stimulated with HA class II or HA class I peptides, or non-stimulated. (D) Frequency of IFNγ-secreting cell per million cells after HA class II (left) and HA class I stimulation (right). Sidak’s multiple comparisons test was used for B and D. Adjusted p-values are indicated. Source data are provided as a Source Data file.

**Supplementary tables:**

**Supplementary Table 1**. Clinical and biological characteristics of patients with AIH and NASH expressed as mean [95% confidence interval] in the figure 5.

| Group | Active AIH (AIHa) | Remission AIH (AIHr) | NASH |
| --- | --- | --- | --- |
|  | n=14 | n=13 | n=5 |
| Age (year) | 50,3 [39,7-61] | 52,5 [39,1-65,8] | 59,2 [33,3-85,1] |
| Female, n (%) | 9 (64%) | 9 (69%) | 3 (60%) |
| IgG (g/L) | 19,5 [15,2-23,8] | 11,4 [9,9-12,9] | 11,9 [6,8-17,1] |
| AST (IU/L) | 307 [121,7-492,1] | 22,6 [17,4-27,9] | 45,9 [23,4-68,4] |
| ALT (IU/L) | 505 [185,6-824,7] | 20,8 [14,4-27,1] | 73,5 [6,2-140,9] |
| Immunosuppressive agents n (%) |  |  |  |
| No, n (%) | 9 (64%) | 0 | 5 (100%) |
| Naïve | 9 | 0 | NA |
| Yes, n (%) | 5 (36%) | 13 (100%) | 0 |
| Azathioprin | 4 | 11 | NA |
| MMF | 1 | 1 | NA |
| Steroids only | 0 | 1 | NA |
| Steroids associated to other therapy | 2 | 3 | NA |

Clinical and biological characteristics of patients with AIH and NASH expressed as mean [95% confidence interval].

AIH: Autoimmune Hepatitis, NASH: Non Alcoholic Steatohepatitis, AST: Aspartate Aminotransferase, ALT: Alanine Aminotransferase, NA: non-applicable

**Supplementary Table 2.** Antibody listing.

| **Name** | **Clone** | **Antigen** | **Brand** | **reference** | **Dilution** | **Reactivity** | **Application** |
| --- | --- | --- | --- | --- | --- | --- | --- |
| APC/Cyanine7 anti-human CD69 | FN50 | CD69 | BioLegend | 310914 | 20 | Human | Flowcytometry |
| Alexa Fluor647 anti-human CD185 (CXCR5) | J252D4 | CD185 | BioLegend | 356906 | 20 | Human | Flowcytometry |
| PerCP/Cyanine5.5 anti-human CD279 (PD-1) | EH12.2H7 | CD279 | BioLegend | 329914 | 20 | Human | Flowcytometry |
| Alexa Fluor 488 anti-human CD4 | OKT4 | CD4 | BioLegend | 317420 | 20 | Human | Flowcytometry |
| APC/Cyanine7 anti-human CD4 | RPA-T4 | CD4 | BioLegend | 300518 | 20 | Human | Flowcytometry |
| APC anti-human CD25 | BC96 | CD25 | Invitrogen | 17-0259-42 | 20 | Human | Flowcytometry |
| PerCP/Cyanine5.5 anti-human HLA-DR | G46-6 | HLA-DR | BD Biosciences | 560652 | 20 | Human | Flowcytometry |
| Alexa Fluor 488 anti-human CD185 (CXCR5) | RF8B2 | CD185 | BD Biosciences | 558112 | 20 | Human | Flowcytometry |
| Brilliant Violet 421 anti-human CD45RA | HI100 | CD45RA | BioLegend | 304130 | 20 | Human | Flowcytometry |
| APC anti-human CD184 (CXCR4) | 12G5 | CD184 | BioLegend | 306510 | 20 | Human | Flowcytometry |
| Brilliant Violet 421 anti-human CD278 (ICOS) | C398.4A | CD278 | BioLegend | 313524 | 20 | Human | Flowcytometry |
| Brilliant Violet 605 anti-human CD200 (OX2) | OX-104 | CD200 | BioLegend | 329218 | 20 | Human | Flowcytometry |
| Brilliant Violet 650 anti-human CD183 (CXCR3) | G025H7 | CD183 | BioLegend | 353730 | 20 | Human | Flowcytometry |
| Brilliant Violet 711 anti-human CD196 (CCR6) | G034E3 | CD196 | BioLegend | 353436 | 20 | Human | Flowcytometry |
| Brilliant Violet 750 anti-human CD185 (CXCR5) | J252D4 | CD185 | BioLegend | 356942 | 20 | Human | Flowcytometry |
| Brilliant Violet 785 anti-human CD49d | 9F10 | CD49d | BioLegend | 304344 | 20 | Human | Flowcytometry |
| FITC anti-human CD57 | HNK-1 | CD57 | BioLegend | 359604 | 20 | Human | Flowcytometry |
| PE/Cyanine5 anti-human HLA-DR | L243 | HLA-DR | BioLegend | 307608 | 20 | Human | Flowcytometry |
| PE/Fire 700 anti-human CD25 | M-A251 | CD25 | BioLegend | 356146 | 20 | Human | Flowcytometry |
| PerCP/Cyanine5.5 anti-human CD27 | M-T271 | CD27 | BioLegend | 356408 | 20 | Human | Flowcytometry |
| Alexa Fluor 700 anti-human CD127 (IL-7Rα) | A019D5 | CD127 | BioLegend | 351344 | 20 | Human | Flowcytometry |
| APC/Fire 810 anti-human CD38 | HIT2 | CD38 | BioLegend | 303550 | 20 | Human | Flowcytometry |
| BD OptiBuild BUV395 Mouse Anti-Human CD45RA | 5H9 | CD45RA | BD Biosciences | 740315 | 20 | Human | Flowcytometry |
| BD Horizon BUV496 Mouse Anti-Human CD16 | 3G8 | CD16 | BD Biosciences | 612945 | 20 | Human | Flowcytometry |
| BD Horizon BUV737 Mouse Anti-Human CD56 | NCAM16.2 | CD56 | BD Biosciences | 612766 | 20 | Human | Flowcytometry |
| BD Horizon BUV805 Mouse Anti-Human CD8 | SK1 | CD8 | BD Biosciences | 612889 | 20 | Human | Flowcytometry |
| Brilliant Violet 510 anti-human CD3 | OKT3 | CD3 | BioLegend | 317332 | 20 | Human | Flowcytometry |
| Pacific Blue anti-human CD161 | HP-3G10 | CD161 | BioLegend | 339926 | 20 | Human | Flowcytometry |
| PE anti-human TIGIT (VSTM3) | A15153G | TIGIT | BioLegend | 372704 | 20 | Human | Flowcytometry |
| PE/Cyanine7 anti-human CD279 (PD-1) | EH12.2H7 | CD279 | BioLegend | 329918 | 20 | Human | Flowcytometry |
| Spark Blue 550 anti-human CD4 | SK3 | CD4 | BioLegend | 344656 | 20 | Human | Flowcytometry |
| APC/Fire 750 anti-human/mouse Granzyme B | QA16A02 | Granzyme B | BioLegend | 372210 | 20 | Human | Flowcytometry |
| Brilliant Violet 650 anti-human CD28 | CD28.2 | CD28 | BioLegend | 302946 | 20 | Human | Flowcytometry |
| Brilliant Violet 711 anti-human CD39 | A1 | CD39 | BioLegend | 328228 | 20 | Human | Flowcytometry |
| Brilliant Violet 785 anti-human CD27 | O323 | CD27 | BioLegend | 302832 | 20 | Human | Flowcytometry |
| BD Horizon PE-CF594 Mouse Anti-EOMES | X4-83 | EOMES | BD Biosciences | 567167 | 20 | Human | Flowcytometry |
| Spark NIR 685 anti-human FOXP3 | 206D | FOXP3 | BioLegend | 320130 | 20 | Human | Flowcytometry |
| Anti-Hu CD20-BX007—Alexa Fluor 750 | L26 | CD20 | Akoya | 4450018 | 200 | Human | Phenocycler |
| Anti-Hu CD31-BX001—Alexa Fluor 750 | EP3095 | CD31 | Akoya | 4450017 | 200 | Human | Phenocycler |
| Anti-Hu Pan-Cytokeratin-BX019—Alexa Fluor 750 | AE1-AE3 | PANCK | Akoya | 4450020 | 200 | Human | Phenocycler |
| Anti-Hu CD8-BX026—Atto 550 | C8/144B | CD8 | Akoya | 4250012 | 200 | Human | Phenocycler |
| Anti-Hu CD21-BX032—Atto 550 | EP3093 | CD21 | Akoya | 4450027 | 200 | Human | Phenocycler |
| Anti-Hu CD38-BX089—Atto 550 | AKYP0110* | CD38 | Akoya | 4550112 | 200 | Human | Phenocycler |
| Anti-Hu IFNG-BX020—Atto 550 | AKYP0093* | IFNG | Akoya | 4250062 | 100 | Human | Phenocycler |
| Anti-Hu PD-1-BX046—Cy5 | NAT105 | CD279 | Akoya | 4550038 | 200 | Human | Phenocycler |
| Anti-Hu ICOS-BX054—Cy5 | SP98 | ICOS | Akoya | 4350059 | 100 | Human | Phenocycler |
| Anti-Hu CD4-BX003—Cy5 | EPR6855 | CD4 | Akoya | 4350018 | 100 | Human | Phenocycler |
| Anti-Hu CD3e-BX045—Cy5 | EP449E | CD3e | Akoya | 4450030 | 200 | Human | Phenocycler |
| Anti-Hu CXCR5-BX042—Cy5 | EPR23463-30 | CXCR5 | Abcam | ab272936 | 50 | Human | Phenocycler |
| PerCP/Cyanine5.5 anti-mouse CD4 | RM4-5 | CD4 | BD Biosciences | 550954 | 200 | Mouse | Flowcytometry |
| FITC anti-mouse CD4 | GK1.5 | CD4 | BioLegend | 100406 | 200 | Mouse | Flowcytometry |
| FITC anti-mouse CD8a | 53-6.7 | CD8 | BD Biosciences | 553031 | 200 | Mouse | Flowcytometry |
| APC anti-mouse CD44 | IM7 | CD44 | BioLegend | 559250 | 200 | Mouse | Flowcytometry |
| APC/Cyanine7 anti-mouse CD44 | IM7 | CD44 | BioLegend | 103027 | 200 | Mouse | Flowcytometry |
| Brilliant Violet 421 anti-mouse CD279 (PD-1) | J43 | CD279 | BD Biosciences | 562584 | 200 | Mouse | Flowcytometry |
| Brilliant Violet 510 anti-mouse CD19 | 1D3 | CD19 | BioLegend | 115545 | 200 | Mouse | Flowcytometry |
| InVivoMAb anti-mouse PD-1 (CD279) | 29F.1A12 | CD279 | BioXCell | BE0273 | See methods | Mouse | Blockade |
| InVivoMAb anti-mouse CTLA-4 (CD152) | 9H10 | CD152 | BioXCell | BE0131 | See methods | Mouse | Blockade |
| Ultra-LEAF Purified Rat IgG2a,κ isotype control | RTK2758 | / | Biolegend | 400565 | See methods | Mouse | Blockade (control) |
| InVivoMAb polyclonal Syrian hamster IgG | ref BE0087 | / | BioXCell | BE0087 | See methods | Mouse | Blockade (control) |
| InVivoMAb anti-mouse CD4 | GK1.5 | CD4 | BioXCell | BE0003-1 | See methods | Mouse | Depletion |
| InVivoMAb rat IgG2b,κ isotype control | LTF-2 | / | BioXCell | BE0090 | See methods | Mouse | Depletion (control) |

**Supplementary Table 3:** Oligonucleotides listing.

| Name | Forward/Reverse | Sequence (5’-3’) | Provider | Application |
| --- | --- | --- | --- | --- |
| Cre-F | Forward | CCTGGAAAATGCTTCTGTCCG | Eurofins Genomics | PCR (mouse genotyping) & qPCR |
| Cre-R | Reverse | CAGGGTGTTATAAGCAATCCC | Eurofins Genomics | PCR (mouse genotyping) & qPCR |
| HA-F | Forward | AAACTCTTCGCGGTCTTTCCA | Eurofins Genomics | qPCR |
| HA-R | Reverse | GATAAGGTAGCTTGGGCTGC | Eurofins Genomics | qPCR |
| ACTB-F | Forward | TACCACAGGCATTGTGATGG | Eurofins Genomics | qPCR |
