# Supplementary material for: Single cell profiling of circulating autoreactive CD4 T cells from patients with autoimmune liver diseases suggests tissue imprinting
Source: Nat Commun. 2025 Jan 29;16:1161. doi: 10.1038/s41467-025-56363-2 (PMC11779892; doi:10.1038/s41467-025-56363-2)
Supplement: Supplementary file 2 — Description of Additional Supplementary Files [file 41467_2025_56363_MOESM2_ESM.pdf]

Description of Additional Supplementary Files

File Name: **Supplementary Data 1**
Description: Clinical and biological characteristics of patients with AILD presented in the Figure 1.

File Name: **Supplementary Data 2**
Description: List of the significant genes per cluster identified in the figure 2F.

File Name: **Supplementary Data 3**
Description: GO biological process genes linked to the data in the figure 2F and in the figure 6.

File Name: **Supplementary Data 4**
Description: List of genes used for the gene set score analysis.

File Name: **Supplementary Data 5**
Description: P values of the gene set score analysis (Supplementary Figure 4)

File Name: **Supplementary Data 6**
Description: List of the liver clonotypes (TCR beta sequences).

File Name: **Supplementary Data 7**
Description: List of the blood CD4 T cell clonotypes (TCR beta sequences).

File Name: **Supplementary Data 8**
Description: List of the significant genes per cluster identified in the figure 3.

File Name: **Supplementary Data 9**
Description: P values of the gene set score analysis (Figure 3E and supplementary Figure 12).

File Name: **Supplementary Data 10**
Description: Genes data presented in the figure 5F.

File Name: **Supplementary Data 11**
Description: List of the significant genes of HA-specific-CD4 T cells from the liver or the spleen (Figure 6G).
