# Supplementary material for: Single cell profiling of circulating autoreactive CD4 T cells from patients with autoimmune liver diseases suggests tissue imprinting
Source: Nat Commun. 2025 Jan 29;16:1161. doi: 10.1038/s41467-025-56363-2 (PMC11779892; doi:10.1038/s41467-025-56363-2)
Supplement: Supplementary file 14 — Reporting Summary [file 41467_2025_56363_MOESM14_ESM.pdf]

nature portfolio

Corresponding author(s): Sophie Conchon, Pierre Milpied and Amédée Renand

Last updated by author(s): Jan 13, 2025

Reporting Summary

Nature Portfolio wishes to improve the reproducibility of the work that we publish. This form provides structure for consistency and transparency in reporting. For further information on Nature Portfolio policies, see our Editorial Policies and the Editorial Policy Checklist.

Statistics

For all statistical analyses, confirm that the following items are present in the figure legend, table legend, main text, or Methods section.

n/a	Confirmed	
☐	☒	The exact sample size (*n*) for each experimental group/condition, given as a discrete number and unit of measurement
☐	☒	A statement on whether measurements were taken from distinct samples or whether the same sample was measured repeatedly
☐	☒	The statistical test(s) used AND whether they are one- or two-sided<br>*Only common tests should be described solely by name; describe more complex techniques in the Methods section.*
☒	☐	A description of all covariates tested
☐	☒	A description of any assumptions or corrections, such as tests of normality and adjustment for multiple comparisons
☐	☒	A full description of the statistical parameters including central tendency (e.g. means) or other basic estimates (e.g. regression coefficient) AND variation (e.g. standard deviation) or associated estimates of uncertainty (e.g. confidence intervals)
☐	☒	For null hypothesis testing, the test statistic (e.g. *F*, *t*, *r*) with confidence intervals, effect sizes, degrees of freedom and *P* value noted<br>*Give P values as exact values whenever suitable.*
☒	☐	For Bayesian analysis, information on the choice of priors and Markov chain Monte Carlo settings
☒	☐	For hierarchical and complex designs, identification of the appropriate level for tests and full reporting of outcomes
☒	☐	Estimates of effect sizes (e.g. Cohen's *d*, Pearson's *r*), indicating how they were calculated

Our web collection on statistics for biologists contains articles on many of the points above.

Software and code

Policy information about availability of computer code

Data collection	Scripts used for analyzing the datasets and producing the figures of this article are available in the following repository: https://github.com/MilpiedLab/Autoreactive-CD4-T-cells-in-liver-disease.
Data analysis	For flow cytometry: FlowJo and Omiq software and FlowSOM algorithm; for scRNA-seq: Seurat v4.1.0 package in R v4.1.2 or v4.2.2; for mini-bulk-RNA-seq: DEseq2 package; for Bulk TCRBeta seq: RStudio (version 1.1.456) and the tcR, ade4, and tidyverse packages; for spatial multi-phenotyping: Cellpose algorithm and CODEX MAV software; for 3'-end bulk RNA sequencing: R (v4.2.3), limma package; for statistical analysis: GraphPad Prism software V.5 and FaDA (https://shiny-bird.univ-nantes.fr/app/Fada).

For manuscripts utilizing custom algorithms or software that are central to the research but not yet described in published literature, software must be made available to editors and reviewers. We strongly encourage code deposition in a community repository (e.g. GitHub). See the Nature Portfolio guidelines for submitting code & software for further information.

Data

Policy information about availability of data

All manuscripts must include a data availability statement. This statement should provide the following information, where applicable:

- Accession codes, unique identifiers, or web links for publicly available datasets
- A description of any restrictions on data availability
- For clinical datasets or third party data, please ensure that the statement adheres to our policy

All data are included in the Supplementary Information or available from the authors, as are unique reagents used in this Article. The raw numbers for charts and

graphs are available in the Source Data file whenever possible. Source data are provided with this paper. The FCS data (Figure 5) are available under restricted access due to unpublished and ongoing analysis, access can be obtained upon request within a reasonable timeframe after publication. Single-cell RNA sequencing raw data from the different experiments analyzed in this article have been deposited in the NCBI GEO repository under accession numbers GSE270739, GSE269661 and GSE269525. Processed and annotated single-cell RNA-sequencing datasets analyzed in this article are available in the following repository: https://zenodo.org/records/14516943.

Research involving human participants, their data, or biological material

Policy information about studies with human participants or human data. See also policy information about sex, gender (identity/presentation), and sexual orientation and race, ethnicity and racism.

Reporting on sex and gender	All the patients eligible signed a written informed consent prior to inclusion into a bio-bank of samples of AILD patients (BIO-MAI-FOIE). In this study, neither sex nor gender was taken into account. No sex analysis was carried out, as AILD are rare diseases that predominantly affects womenNevertheless, sex was indicated in the clinical data.
Reporting on race, ethnicity, or other socially relevant groupings	Reports of race, ethnicity or other social factors are not recorded or used as variables. As autoimmune liver disease is a rare disease, all clinically eligible patients are potentially included.
Population characteristics	All AIH patients included in this study had a simplified diagnostic score superior or equal to 6 according to the simplified scoring system for AIH of the international autoimmune hepatitis group (IAHG). PBC patients included in this study were diagnose based on cholestasis, increase serum IgM and presence of anti-mitochondria M2 (anti-M2) antibody. Active AIH (AIHa) patients are untreated patients with new onset AIH patients enrolled at diagnosis prior any treatment initiation, and AIH patients under standard treatment but do not normalize the transaminases (AST and ALT) and/or the serum IgG levels or are under relapsing event. Remission AIH (AIHr) patients are defined biochemically by a normalization of the transaminases and the IgG levels, according to the most recent European clinical practice guidelines on AIH. All NASH (non-Alcoholic SteatoHepatitis) patients had histological evidence of NASH and had dysmetabolic syndromes. Sex, age and clinical charcteristics are listed in supplementary tables 1 and 10.
Recruitment	AIH patients with anti-SLA antibodies, anti-LKM1 antibodies or without anti-SLA nor anti-LKM1 antibodies. Active AIH patients and Remission AIH patients. PBC patients with anti-M2 antibodies. NASH patients.
Ethics oversight	COMITE DE PROTECTION DES PERSONNES OUEST IV-NANTES CPP; Ministère de la Recherche, ref MESR DC-2017-2987

Note that full information on the approval of the study protocol must also be provided in the manuscript.

Field-specific reporting

Please select the one below that is the best fit for your research. If you are not sure, read the appropriate sections before making your selection.

☒ Life sciences ☐ Behavioural & social sciences ☐ Ecological, evolutionary & environmental sciences

For a reference copy of the document with all sections, see nature.com/documents/nr-reporting-summary-flat.pdf

Life sciences study design

All studies must disclose on these points even when the disclosure is negative.

Sample size	No sample size was calculated. AIH or PBC patients with anti-SLA (n=12), anti-LKM1 (n=4) or anti-M2 (n=14) antibodies are rare; groups were formed with all available patients in the cohort. Biopsies and matched blood samples for patients with AIH at diagnosis are limited (; samples from four separate patients were used. Patients with active AIH are also rare, as newly diagnosed patients are rapidly treated. For mouse experiments, a minimum of four mice per group was used (to limit the use of animals).
Data exclusions	no data exclusions
Replication	Technical replication was used only for in vitro assays (TCR stimulation, Figure 5F), Elisa assays (Figure 6, figure 7 and supplementary figure 19), Elispot assays (Supplementary Figure 21) and RT-PCR methods (Figure 6, and supplementary figure 19), and is presented as the mean value (of the three replicates) per biological replicate (sample). The protocols for the mouse experiments were also repeated at least three times, and all points in the figures represent a biological replicate.
Randomization	Patients in the different groups described above were randomly selected during the course of the study.
Blinding	Blinding was not possible due to the need to select patients according to their clinical outcome. For mouse experiments the investigators were blinded to group allocation during data collection and analysis.

Reporting for specific materials, systems and methods

We require information from authors about some types of materials, experimental systems and methods used in many studies. Here, indicate whether each material, system or method listed is relevant to your study. If you are not sure if a list item applies to your research, read the appropriate section before selecting a response.

Materials & experimental systems

n/a	Involved in the study
☐	☒ Antibodies
☒	☐ Eukaryotic cell lines
☒	☐ Palaeontology and archaeology
☐	☒ Animals and other organisms
☒	☐ Clinical data
☒	☐ Dual use research of concern
☒	☐ Plants

Methods

n/a	Involved in the study
☒	☐ ChIP-seq
☐	☒ Flow cytometry
☒	☐ MRI-based neuroimaging

Antibodies

Antibodies used	Supplementary Table 2 in the manuscript
Validation	All primary antibodies have been titrated and validated according to the manufacturer's recommendations.

Animals and other research organisms

Policy information about studies involving animals; ARRIVE guidelines recommended for reporting animal research, and Sex and Gender in Research

Laboratory animals	Heterozygous transthyretin (TTR)-inducible Cre (iCre) mice, originally on a C57Bl6 backgroud, were back-crossed on a Balb/c background for at least 10 generations (TAAM, CDTA CNRS Orléans, FRANCE). They were cross-bred with homozygous Rosa26 hemagglutinin (HA) floxed mice (Rosa26tm(HA)1Libl, Kindly provided by R. Liblau, Toulouse, France), resulting in heterozygous Rosa26 HA floxed (Ctrl) mice and Rosa26 HA floxed TTR-inducible Cre (HA/iCre) mice.Male and female eight to twelve-weeks-old mice were used for each experiment
Wild animals	The study did not invovled wild animals
Reporting on sex	no sex-based analysis was carried out. Approximately the same number of male and female animals were used in each group. Male and female eight to twelve-weeks-old mice were used for each experiment.
Field-collected samples	All mice were housed at the UTE IRS-UN animal facilities (Nantes, FRANCE) in specific pathogen-free conditions. Mice were housed at a maximum of five mice per individually ventilated cage in 12h:12h light/dark cycle and under standard conditions of temperature (21-24°C) and humidity (40-60%). Mice were fed ad libitum with continuous access to tap water. At the end of experiments, mice were anaesthetized and euthanized by cervical dislocation.
Ethics oversight	Procedures were approved by the regional ethical committee for animal care (Comité d'éthique de l'expérimentation animale des Pays de la Loire (CEEA-Pdl)) and by the Ministère de l'enseignement supérieur et de la recherche (agreements APAFIS #2054, #28582 and #43529).

Note that full information on the approval of the study protocol must also be provided in the manuscript.

Plants

Seed stocks	*Report on the source of all seed stocks or other plant material used. If applicable, state the seed stock centre and catalogue number. If plant specimens were collected from the field, describe the collection location, date and sampling procedures.*
Novel plant genotypes	*Describe the methods by which all novel plant genotypes were produced. This includes those generated by transgenic approaches, gene editing, chemical/radiation-based mutagenesis and hybridization. For transgenic lines, describe the transformation method, the number of independent lines analyzed and the generation upon which experiments were performed. For gene-edited lines, describe the editor used, the endogenous sequence targeted for editing, the targeting guide RNA sequence (if applicable) and how the editor was applied.*
Authentication	*Describe any authentication procedures for each seed stock used or novel genotype generated. Describe any experiments used to assess the effect of a mutation and, where applicable, how potential secondary effects (e.g. second site T-DNA insertions, mosiacism, off-target gene editing) were examined.*

Flow Cytometry

Plots

Confirm that:

- [x] The axis labels state the marker and fluorochrome used (e.g. CD4-FITC).
- [x] The axis scales are clearly visible. Include numbers along axes only for bottom left plot of group (a 'group' is an analysis of identical markers).
- [x] All plots are contour plots with outliers or pseudocolor plots.
- [x] A numerical value for number of cells or percentage (with statistics) is provided.

Methodology

Sample preparation	Human PBMCs: after isolation by ficoll, and after in vitro or non-in vitro stimulation, PBMCs were stained with appropriate antibodies. Extraction of mouse cells : Splenocytes were isolated by mechanical dissociation of spleen in red blood cell lysis buffer; Liver non-parenchymal cells (NPCs) were isolated after in vivo elimination of blood by perfusion of HBSS buffer. Livers were digested with collagenase IV, and NPCs enriched by Percoll density gradient centrifugation and red blood cells lysis .
Instrument	BD FACSCantoII, Cytek Aurora and BD FACSAriaII
Software	FlowJo and Omiq software
Cell population abundance	post-sorting purity is determined by rapid acquisition of the collection tube at the end of cell sorting. Sorting is considered valid when the purity of the sorted population exceeds 85%.
Gating strategy	FSC-A/SSC-A: lymphocyte gating; FSC-W/SSC-W: single cell gating; CD3/live staining with or without dump channel: live cell gating; CD3+CD4+ gating: focus on CD4 T cells for analysis.

- [x] Tick this box to confirm that a figure exemplifying the gating strategy is provided in the Supplementary Information.
